# BCAS2, a protein enriched in advanced prostate cancer, interacts with NBS1 to enhance DNA double-strand break repair

**DOI:** 10.1038/s41416-020-01086-y

**Published:** 2020-09-23

**Authors:** Li-Po Wang, Tzu-Yu Chen, Chun-Kai Kang, Hsiang-Po Huang, Show-Li Chen

**Affiliations:** 1 Graduate Institute of Microbiology, College of Medicine, National Taiwan University, Taipei, Taiwan; 2 Graduate Institute of Medical Genomics and Proteomics, College of Medicine, National Taiwan University, Taipei, Taiwan

**Keywords:** Double-strand DNA breaks, Prostate cancer

## Abstract

**Background:**

Breast cancer amplified sequence 2 (BCAS2) plays crucial roles in pre-mRNA splicing and androgen receptor transcription. Previous studies suggested that BCAS2 is involved in double-strand breaks (DSB); therefore, we aimed to characterise its mechanism and role in prostate cancer (PCa).

**Methods:**

Western blotting and immunofluorescence microscopy were used to assay the roles of BCAS2 in the DSBs of PCa cells and apoptosis in *Drosophila*, respectively. The effect of BCAS2 dosage on non-homologous end joining (NHEJ) and homologous recombination (HR) were assayed by precise end-joining assay and flow cytometry, respectively. Glutathione-S-transferase pulldown and co-immunoprecipitation assays were used to determine whether and how BCAS2 interacts with NBS1. The expression of BCAS2 and other proteins in human PCa was determined by immunohistochemistry.

**Results:**

BCAS2 helped repair radiation-induced DSBs efficiently in both human PCa cells and *Drosophila*. BCAS2 enhanced both NHEJ and HR, possibly by interacting with NBS1, which involved the BCAS2 N-terminus as well as both the NBS1 N- and C-termini. The overexpression of BCAS2 was significantly associated with higher Gleason and pathology grades and shorter survival in patients with PCa.

**Conclusion:**

BCAS2 promotes two DSB repair pathways by interacting with NBS1, and it may affect PCa progression.

## Background

In cells, DNA double-strand breaks (DSB) are a major type of stress during DNA replication or attacks by reactive oxygen species, chemicals, and physical agents, such as UV light and ionising radiation (IR). Incorrectly repaired DSBs can lead to serious consequences, including cell apoptosis and carcinogenesis.^1,2^ There are two major forms of DNA DSB repair in eukaryotes: homologous recombination (HR) and non-homologous end joining (NHEJ).^3^ In HR, broken ends are processed by proteins, including BLM, CtIP and the MRE11-RAD50-NBS1 (MRN) complex, to generate 3′-single-stranded DNA (ssDNA) overhangs, which are protected from nucleases by replication protein A (RPA). They are transformed into RAD51-ssDNA nucleoprotein filaments that participate in the formation of D-loop structures and facilitate synapse formation with template homologous sequences for DNA repair.^4^ In the eukaryote NHEJ pathway, Ku proteins bind to DNA break ends and recruit the DNA–protein kinase catalytic subunit to juxtapose them, which then recruits nucleases, polymerase and ligases to fix the damaged sites, finally collaborating with the XRCC4/Ligase4/XLF complex to resolve and fix the broken ends.^5^

The MRN complex plays crucial roles in both DNA repair and checkpoint activation.^6^ Upon DSB induction, the MRN complex binds to free DNA ends and induces the active form of ATM, which phosphorylates γ-H2AX in the nearby DSB-flanking chromatin.^7,8^ Among the MRN complex proteins, NBS1 plays the roles of recruiter and coordinator in the rapid assembly of the MRN complex at damaged sites. NBS1 is also essential for the nuclear localisation and adequate performance of MRE11 and RAD50. Although NBS1 has no enzymatic activity or DNA-binding ability, it contains a forkhead-associated domain and two adjacent BRCA1 C-terminal domains in its N-terminus. The C-terminus of NBS1 is able to interact with phosphatidylinositol 3-kinase-related kinases, such as ATM and ATR.^9,10^

Breast carcinoma-amplified sequence 2 (BCAS2) is a member of the spliceosome complex, which also includes the PSO4, CDC5L and PLRG1 proteins. In addition to their roles in pre-mRNA splicing, these proteins also have important functions in DNA repair,^11–13^ cell-cycle control, apoptosis and adult tissue homoeostasis.^14^ The cellular functions of BCAS2 are of special interest. We previously demonstrated that BCAS2 is a negative regulator of p53, which is involved in cell-cycle arrest and apoptosis.^15^ BCAS2 also enhances androgen receptor mRNA transcription and protein stability through forming a complex with the HSP90-androgen receptor, thus promoting the proliferation of prostate cancer (PCa) cells.^16^ We also demonstrated that BCAS2 contributes to pre-mRNA splicing.^17^ It directly binds with CDC5L and recruits the PSO4 complex, and it is vital in the viability of *Drosophila*.^17^ In addition, *Drosophila* BCAS2 participates in *Delta* pre-mRNA splicing, which regulates Delta-Notch signalling in *Drosophila* wing development.^18^ BCAS2 has other physiological roles, such as acting as a negative regulatory factor in heat-shock factor 4 protein homoeostasis.^19^ Because it interacts with the RPA complex and modulates ATR activation during DNA repair,^20^ it is essential in maintaining genome integrity in mouse early embryos.^21^

In this study, we investigate the roles of BCAS2 in DSB repair caused by IR using in vitro and in vivo models. Our results demonstrate that BCAS2 enhances the efficiency of both HR and NHEJ and interacts with NBS1 through a specific domain. In addition, we propose that BCAS2 has a potential role in tumour progression based on the correlation of the expression levels of BCAS2 and its downstream effector β-catenin with clinical and pathological parameters.

## Methods

The full descriptions of materials and methods used in this study are provided in Supplementary Materials and Methods.

### In vitro NHEJ assay

HEK 293T cells were cultured in large quantities, and the lysates underwent subcellular fractionation to obtain a nuclear pellet, which was then dialysed. The pBSK (+) duplex plasmid DNA was linearised with *EcoR*I and then co-incubated with nuclear extracts, in which genomic DNAs were fragmented into small pieces (<500 bp) by sonication, in reaction buffer at 37 °C for 1 h according to previously described methods^22,23^ and then subjected to 0.7% agarose gel electrophoresis after deproteinisation (10 μg/μl proteinase K, 3% SDS, 50 mM EDTA and 100 mM Tris-HCL, pH 7.5, 90 min at 57 °C). The gel was then incubated with the highly sensitive nucleic acid dye GelRed (Sigma-Aldrich) at 1:10,000 dilution in water at room temperature for 15 min and then de-stained in water for ~1 h. The Quantification of the DNA bands was performed using a phosphorimager (Analytik Jena AG).

### In vivo NHEJ assay

Plasmid pGL3 was linearised by the restriction enzymes *Sph*I or *Hind* III, followed by DNA clean-up using a PCR Purification Kit (Qiagen). It was then examined by agarose gel electrophoresis. Linear pGL3 and internal control DNA (Renilla luciferase reporter) were transiently co-transfected to HEK 293T cells using the FuGENE HD Transfection Reagent (Promega). After 48 h, the transfectants were harvested and analysed for luciferase activity using a Dual-Luciferase Reporter kit (Promega), as previously described.^16^

### Homologous recombination assay

Transfection mixtures including plasmid DNA pmHPRT-DR green fluorescent protein (GFP), pCBAsce vectors^24^ (encoding the restriction enzyme I-SceI) and pDSRed (an internal control for correcting transfection efficiency), were delivered into HEK 293T cells using lipofectamine 2000 (Invitrogen). The cells were suspended, and the light signals were sorted by flow cytometry after 48 h; only the cells that emitted dual green/red fluorescence dyes were considered positive.

### Immunohistochemistry and scores of protein expressions

Human prostate specimens were purchased from US Biomax (serial numbers: PR753 and PR483b, Rockville, MD, USA) and obtained from the National Taiwan University (NTU) Hospital Tissue Bank, respectively. The study involving human tissues was approved by the Institutional Review Boards at NTU. The tissue slides were de-paraffinised in xylene, rehydrated and then boiled in citrate buffer to achieve antigen retrieval. The tissues were then subjected to permeabilisation and blocking according to the manufacturer’s protocols for the immunohistochemistry (IHC) kits (BioGenex, QD420-YIKE). The specimens were then incubated with anti-β-catenin or anti-BCAS2 antibodies. Biotinylated secondary antibodies were used to detect primary antibody–antigen complexes in the samples, followed by incubation with streptavidin peroxidase conjugate and 3, 3′-diaminobenzidine for colour development. The staining time of Gill’s Haematoxylin (Sigma-Aldrich) was decreased from 3 to 1 min to avoid the masking effect of nuclear haematoxylin staining on the IHC intensity in the nucleus. The results were observed under a microscope and then assessed by multiplying the intensity scores and the extent scores, which were taken in a double-blind manner by two different researchers with pathology training. The intensity was scored from zero to three, where 0 represented no staining (negative), 1 represented weak staining, 2 represented intermediate staining and 3 represented strong staining. The extent score was calculated by the percentage (0–100%) of all prostate gland areas on the slides of patient specimens. The total score, from 0 to 300, was obtained by multiplying intensity by extent. IHC scores equal to or higher than 150 were defined as high expressions of BCAS2 and β-catenin staining. All the antibodies used are listed in Supplementary Table 1.

### Statistics

Mann–Whitney *U* tests (two-tailed, unpaired) were used to evaluate the differences between the two groups. Kruskal–Wallis tests with post hoc multiple comparisons (the Dunn’s test) were used to compare the differences among multiple groups. Survival analysis was performed by the Kaplan–Meier method, and statistical differences were calculated by the log-rank test. The Spearman’s correlation coefficient was used to assess the strength of a link between two sets of data (IHC scores or mRNA levels). The above analyses were performed using GraphPad Prism 6.0 (GraphPad Software, CA, USA) and MedCalc (MedCalc Software, Ostend, Belgium). *P* values <0.05 were considered statistically significant.

## Results

### BCAS2 enhances the efficiency of DNA repair after ionising radiation-induced DNA damage in human PCa cells

**Fig. 1:**
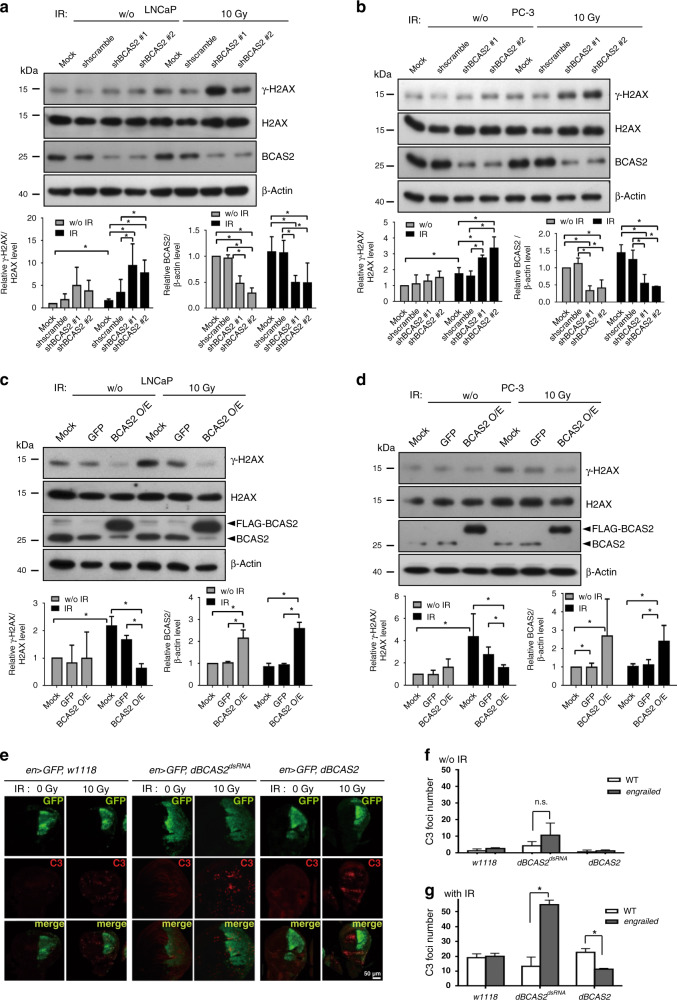
BCAS2 expression mitigated ionising radiation (IR)-induced damage and apoptosis in human prostate cancer (PCa) cells and *Drosophila*, respectively. **a**, **b** Knockdown of BCAS2 expression in human PCa cells increased their damage by IR. Cells with BCAS2 knockdown exhibited an increase in the level of the IR-induced γ-H2AX. LNCaP (**a**) and PC-3 (**b**) cells were infected with lentiviruses containing shRNA for BCAS2 (shBCAS2 #1 and #2) or scrambled shRNA (shscramble), or mock-infected, and then were exposed to a total dose of 10 Gy γ-radiation (dose rate = 3.69 Gy/min); cells were allowed to recover for 8 h before harvest for western blotting. The intensities of γ-H2AX and BCAS2 western blot signals were quantified and normalised to those of the control (total H2AX and β-actin). The histograms (left lower panels) show a significantly increased (*n* = 4; *P < 0.05; Mann–Whitney *U* test) level of γ-H2AX in cells with the downregulation of BCAS2. **c**, **d** PCa cells with the overexpression of BCAS2 had lower levels of IR-induced DNA damage. LNCaP (**c**) and PC-3 (**d**) cells were infected with FLAG-BCAS2- or GFP-containing lentiviruses four days before exposure to 10 Gy γ-radiation; the cells were allowed to recover for 8 h before harvesting for western blotting. The γ-H2AX levels and BCAS2 (the sum of both exogenous FLAG-tagged and endogenous BCAS2) were measured and compared, as described above. Significantly depressed γ-H2AX levels were found in cells overexpressing BCAS2 compared with cells overexpressing GFP (*n* = 4 for **c**; *n* = 3 for **d**; *P < 0.05; Mann–Whitney *U* test). **e**–**g** BCAS2 expression alleviated the severity of IR-induced apoptosis in *Drosophila*. **e** Apoptotic cells in wing discs were visualised and quantified by immunofluorescence microscopy using an anti-cleaved caspase-3 (C3) antibody after exposure to 10 Gy γ-radiation followed by a 6-h recovery period. The signals of *engrailed-Gal4* driven GFP (green) were located at the posterior region of wing discs from wandering third-instar larvae, where *UAS-dBCAS2*^*dsRNA*^ (knockdown of BCAS2) and *UAS-dBCAS2* (overexpression of BCAS2) were expressed. The GFP-negative anterior region of the wing discs and the wild-type (*w1118*) served as controls. Anti-C3 staining (red) revealed apoptotic cells in imaginal wing discs. Merge: merged images. Scale bar = 50 μm. **f**, **g** Histograms of the results in (**e**) show comparisons of C3-positive foci numbers in imaginal wing discs without (**f**) or with (**g**) IR. A minor but non-significant increase (2.5-fold, *P* = 0.13) in C3 foci numbers was found in non-irradiated BCAS2-depleted wing discs compared with controls; in contrast, there was a significant increase (4.1-fold, *P* < 0.05) in C3 foci numbers in the irradiated BCAS2-depleted wing discs compared with control wing discs (*n* = 3 per group; ns. non-significant; *P < 0.05, Mann–Whitney *U* test).

To determine whether BCAS2 is involved in the repair of DSBs caused by ionising radiation (IR), we infected two PCa cell lines—the p53 wild-type LNCaP and the p53-deficient PC-3 cells—with either BCAS2 shRNA-containing (shBCAS2 #1 and #2) or control scrambled shRNA-containing (shscramble) lentivirus. We induced the DSBs by exposing them to 10-Gy IR followed by an 8-h recovery period before harvesting them for use in the experiments. Quantification of the western blotting results showed the significant upregulation of the level of γ-H2AX (Fig. 1a, b), which is a DSB biomarker, in cells with the downregulation of BCAS2 mRNA (Supplementary Fig. S1a) and protein levels (Fig. 1a for LNCaP, Fig. 1b for PC-3) by either one of the two shRNAs compared to control groups (mock and shscrambled infection), suggesting that BCAS2 enhanced the efficiency of DSB repair. In contrast, the levels of γ-H2AX in the BCAS2-overexpressing LNCaP (Fig. 1c) and PC-3 cells (Fig. 1d) were downregulated compared to those of the control groups. Therefore, the BCAS2 expression level was negatively correlated with the level of γ-H2AX at this particular time point after IR, which may reflect DNA repair efficacy, thus supporting our hypothesis that BCAS2 enhances the DNA repair efficacy of IR-induced DNA damage.

### BCAS2 expression reduces the cell death of PCa cells treated with DSB-causing chemotherapeutic drugs

Because BCAS2 enhanced the efficiency of DSB repair in human PCa cells, we were interested to know whether BCAS2 expression could help reduce the DSB-related death of PCa cells. We examined this possibility by the measurement of the cell growth kinetics after treating BCAS2-depleted or non-depleted LNCaP and PC-3 cells with doxorubicin^25^ or etoposide,^26^ which are two topoisomerase II inhibitors capable of causing DSBs and apoptosis. After 3 days of treatment with 100 nM doxorubicin or 25 μM etoposide, the LNCaP cells (Supplementary Fig. S1b, left panel) with knockdown of BCAS2 showed significantly decreased viability compared with the control group during incubation for 3 days. We observed the same phenomenon in the PC-3 cells (Supplementary Fig. S1b, right panel), and those depleted of BCAS2 were more sensitive to both drugs. In contrast, the BCAS2-overexpressing LNCaP cells (Supplementary Fig. S1c, left panel) and PC-3 cells (Supplementary Fig. S1c, right panel) had significantly increased numbers of viable cells under treatment with doxorubicin or etoposide compared to those without BCAS2 overexpression. These results suggested that BCAS2 expression might diminish DSB-related death in PCa cells.

### *Drosophila* BCAS2 (dBCAS2) alleviates IR-induced cell apoptosis in *Drosophila* wing discs in a p53-independent manner

To further examine the possibility that BCAS2 reduces cell death caused by DSBs, we used the *Drosophila* system because it is a valuable in vivo model in the study of DNA damage repair (DDR)^27^ and cell proliferation. The pathways of DSB repair and cell-cycle checkpoints in *Drosophila*, which are similar to those in humans, are easily manipulated and observed. Here, we examined the role of dBCAS2 in the apoptosis of wing discs of the third-instar larvae triggered by DSBs after exposure to IR (10 Gy), followed by a 6-h recovery period using immunofluorescence microscopy with anti-cleaved caspase-3 (C3) to reveal apoptotic cells.^17^ We used *engrailed-GAL4* to drive the shRNA for *dBACS2* (*UAS-dBCAS2*^*dsRNA*^, Fig. 1e, middle two columns) and *dBACS2* cDNA (*UAS-dBCAS2*, Fig. 1e, right two columns) specifically in the posterior part of the wing discs where GFP was also simultaneously expressed. In contrast to the anterior part of the wing discs, where no obvious difference was observed, there was a significantly higher number (4.1-fold increase, *P* = 0.0004) of apoptotic foci (Fig. 1e, red foci, Fig. 1f, g) in the *dBCAS2*^*dsRNA*^ wing discs than in the wild-type (*w1118*) after exposure to IR. However, the overexpression of dBCAS2 (Fig. 1e) significantly reduced the number of IR-related cleaved caspase-3-positive foci in the wing discs compared to the wild-type control (Fig. 1f, g). Furthermore, to determine whether the protective effect afforded by BCAS2 against IR-induced cell apoptosis is dependent on its negative regulatory effect on p53,^15^ we constructed a wing-specific driver (*ms1096-GAL4*) in *dmp53* null mutant flies to specifically deplete dBCAS2 (Supplementary Fig. S2, *dBCAS2*^*dsRNA*^). The dBCAS2 knockdown effect was confirmed by immunostaining for Delta (Supplementary Fig. S2), a wing disc-expressing protein that is encoded by a downstream gene of *dBCAS2*.^18^ Immunofluorescence microscopy showed that the number of IR-induced cleaved caspase-3-positive foci in the p53-null and dBCAS2-depleted wing discs was still significantly higher than in the p53-null and dBCAS2 wild-type flies (Supplementary Fig. S2). We also tried to use an antibody to stain γ-H2Av, the *Drosophila* γ-H2AX homologue, but we failed because the antibody was not suitable for immunostaining (data not shown). Collectively, our results suggest that BCAS2 functions in suppressing apoptosis induced by IR in vivo and that this effect is independent of p53 expression.

### BCAS2 enhances in vitro and in vivo NHEJ efficiency in DSB repair

**Fig. 2:**
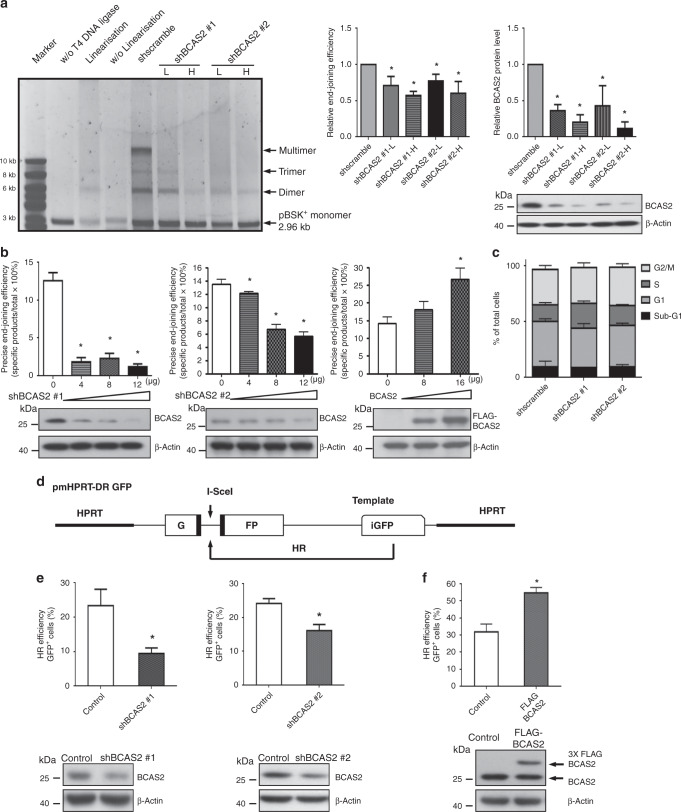
BCAS2 positively regulated DSBs repair by enhancing both non-homologous end joining (NHEJ) and homologous recombination (HR) activity. **a**–**c** The expression level of BCAS2 was positively correlated with NHEJ ability during DSB repair in vitro and in vivo. **a** In vitro precise end-joining assay showed repression of NHEJ activity by the downregulation of BCAS2. Linearised or non-linearised pBSK (+) DNA plasmids were assayed for their degrees of ligation by agarose gel electrophoresis followed by staining with GelRed, a highly sensitive fluorescent nucleic acid dye, after incubation with nuclear extracts from HEK 293T cells infected with lentiviruses with controlled shRNA (shscramble) or shRNAs against BCAS2 (shBCAS2 #1 and #2) in two different titres (L: low titre; H: high titre). The amounts of joined pBSK (+) dimer, trimer and multimer were measured by a phosphorimager, and they represented the degree of NHEJ. End-joining efficiency was calculated as the percentage of oligomers (dimer, trimer, and multimer) in all DNAs (namely, the sum of the oligomers divided by the sum of the monomer and oligomers × 100%). A linearised monomer incubated with T4 DNA ligase served as a positive control; a linearised monomer (linearisation) without T4 DNA ligase treatment and a non-linearised plasmid were negative controls. Data on end-joining efficiency were normalised to those of control (shscramble) and presented as mean plus SD (middle panel; *n* = 3; *P < 0.05; Mann–Whitney *U* test). Quantification of western blotting results (right panel) demonstrated a significant knockdown effect on BCAS2 (*n* = 3; *P < 0.05; Mann–Whitney *U* test). **b** The overexpression and knockdown of BCAS2 showed a dose-dependent activating and repressive effects, respectively, on the precise end-joining activity in HEK 293T cells. The pGL3 plasmid containing the luciferase reporter gene was linearised by *Sph*I / *HindIII* and then co-transfected into HEK 293T cells with an increasing amount of either the plasmid expressing the shRNAs for *BCAS2* (shBCAS2 #1 and #2), or the plasmid expressing the *BCAS2* cDNA tagged with three copies of flag sequence (3× FLAG-BCAS2). The restored bioluminescence activity represented the in vivo-specific NHEJ activity that occurs in the enzyme-digested luciferase coding domain of pGL3 reporter plasmid. The second luciferase (Renilla) reporter plasmid was co-transfected, and it functioned as an internal control to eliminate differences in transfection efficiency between culture wells. The in vivo precise end-joining activity was measured using the Dual-Luciferase Reporter System, as described in “Methods”. The knockdown of BCAS2 (left and middle panels) showed a significant decrease in the precise end-joining activity in HEK 293T cells. The overexpression of BCAS2 (right panel) revealed the opposite effect. Western blotting confirmed the changes in BCAS2 protein level. Data are presented as means + SD (*n* = 3; *P < 0.05, Mann–Whitney *U* test). **c** Flow-cytometry analysis showed no significant differences (Mann–Whitney *U* test) in the phase distribution of the cell cycle between the BCAS2-depleted and control HEK 293T cells. Note that the percentage of cells in the sub-G1 phase may reflect the number of apoptotic cells. **d**–**f** BCAS2 upregulated HR activity. **d** Schematic illustration of the reporter plasmid pmHPRT-DR GFP used for HR assay. A modified fluorescence-based assay was used to assess HR repair by co-transfecting HEK 293T cells with the pmHPRT-DR GFP plasmid, I-SceI endonuclease expression vector pCBASceI and pDsRed plasmid (internal control). The pmHPRT-DR GFP plasmid is composed of two separate, differentially mutated GFP domains, in which the homologous sequences were oriented as direct repeats. The upstream I-SceI-GFP contained the recognition site for the rare-cutting I-SceI endonuclease and two in-frame stop codons, while the downstream sequence enclosed an 812-bp internal GFP fragment (iGFP). When the transfected I-SceI-containing plasmid was expressed and caused a DSB, the downstream internal GFP fragment sequence acted as a donor of the wild-type sequence for the broken I-SceI-GFP gene, which may be repaired through HR and then properly expressed. **e**, **f** The HR repair activity, represented by the number of GFP-positive cells, was then measured by flow cytometry in wild-type, BCAS2-depleted (**e**, shBCAS2 #1 and #2), and BCAS2-overexpressing (**f**, FLAG-BCAS2) HEK 293T cells. The transfection of pDsRed was used to assess transfection efficiency. The efficiency of HR was calculated as a ratio of GFP^+^/DsRed^+^ cells. The changes in BCAS2 protein levels were confirmed by western blotting (lower panels). Data are presented as mean + SD (*n* = 3; *P < 0.05; Mann–Whitney *U* test).

Next, we aimed to determine whether BCAS2 is involved in regulating the two major pathways that repair DNA DSBs: the HR pathway and the NHEJ pathway. To demonstrate that BCAS2 is involved in the NHEJ pathway, we performed a cell-free NHEJ assay as previously reported,^22,23^ in which *Eco*RI-digested pBSK (+) duplex plasmid DNA was incubated with purified HEK 293T nuclear extracts to ligate the DNA. The assay by agarose gel electrophoresis was followed by staining with a highly sensitive DNA dye to detect the re-joined and catenated DNAs (oligomers), which could be separated from the non-re-joined DNAs on the gel because the former migrated more slowly. The results showed that there were significantly fewer re-joined plasmid DNA oligomers in the nuclear extracts from the BCAS2-depleted HEK 293T cells than in those from the knockdown control (Fig. 2a). We further validated the results and measured the changes in NHEJ activity using a well-established cell-based system,^22,28^ in which a reporter plasmid pGL3 linearised by endonuclease *Sph*I was transfected into the HEK 293T cells, and the bioluminescence signal generated by a precise end-joining luciferase gene-containing plasmid was measured to represent NHEJ activity (Fig. 2b). The NHEJ activity in cells co-transfected with shBCAS2 plasmid (either shBCAS2 #1 or #2) was significantly decreased compared to the control, and shBCAS2 #2 plasmid exhibited a dose-dependent effect (Fig. 2b, middle panel). In contrast, the cells co-transfected with the BCAS2 expression plasmid showed a dose-dependent increase in NHEJ activity compared to the cells without BCAS2 overexpression, and they reached a maximum of twofold upregulation (Fig. 2b, right panel). Because cell death and cell-cycle changes may affect DSB repair, we examined such changes in our BCAS2-depleted versus the control HEK 293T cells used in DSB repair experiments. The results (Fig. 2c) showed that neither the apoptosis rate (represented by the percentage of sub-G1 phase) nor the percentage of cells in each cell-cycle phase (G1, S and G2/M) was significantly changed in the cells with BCAS2 knockdown compared to the control cells.

### BCAS2 upregulates the efficiency of homologous recombination during DSB repair

Next, we examined whether BCAS2 participates in HR by co-transfecting HEK 293T cells with the BCAS2-knockdown (shBCAS2 #1, or #2) or BCAS2-overexpression (FLAG-BCAS2) plasmids and the HR reporter plasmid pmHPRT-DR GFP (Fig. 2d).^29^ The enzyme-digested upstream GFP, only when it was correctly repaired in vivo by HR based on the sequence of downstream GFP fragment (Fig. 2d), could be translated into a functional GFP protein and quantified by flow cytometry. The number of GFP-positive cells was significantly diminished (control vs. shBCAS2 #1, 23.3 ± 4.7% vs. 9.5 ± 1.6%, *P* < 0.05) in the BCAS2-depleted HEK 293T cells compared to that in the control cells (Fig. 2e, left panel). The second shBCAS2 construct (shBCAS2 #2) also showed a significant reduction in GFP-positive cell numbers in the cells depleted of BCAS2 compared to the control (control vs. shBCAS2 #2, 24.1 ± 1.4% vs. 16.1 ± 1.8%, *P* < 0.05) (Fig. 2e, right panel). In contrast, in the BCAS2-overexpressing cells, the GFP-positive cell number was significantly higher than in control (control vs. FLAG-BCAS2, 31.8 ± 8.1% vs. 54.7 ± 5.6%, *P* < 0.05) (Fig. 2f). In addition, to evaluate the effect of BCAS2 knockdown on HR efficiency in human cells after IR, we examined the percentage of cells positive for both γ-H2AX and RPA2-pS4/8 foci (the latter as a representative of RPA-coated single-stranded DNA seen in the end processing of HR), in both the HEK 293T and PC-3 cells during a time course after IR (10 Gy) or without IR (control). The results showed that the knockdown of BCAS2 caused a significant reduction in the percentage of γ-H2AX and RPA2-pS4/8 foci double-positive cells in both cell lines at almost each time point of the examination after IR (Supplementary Fig. S3). Together, these results support the hypothesis that BCAS2 plays a positive regulatory role in HR during DSB repair.

### BCAS2 directly interacts with NBS1 through the N-terminus of BCAS2, which is also required for enhancing the efficiency of HR

**Fig. 3:**
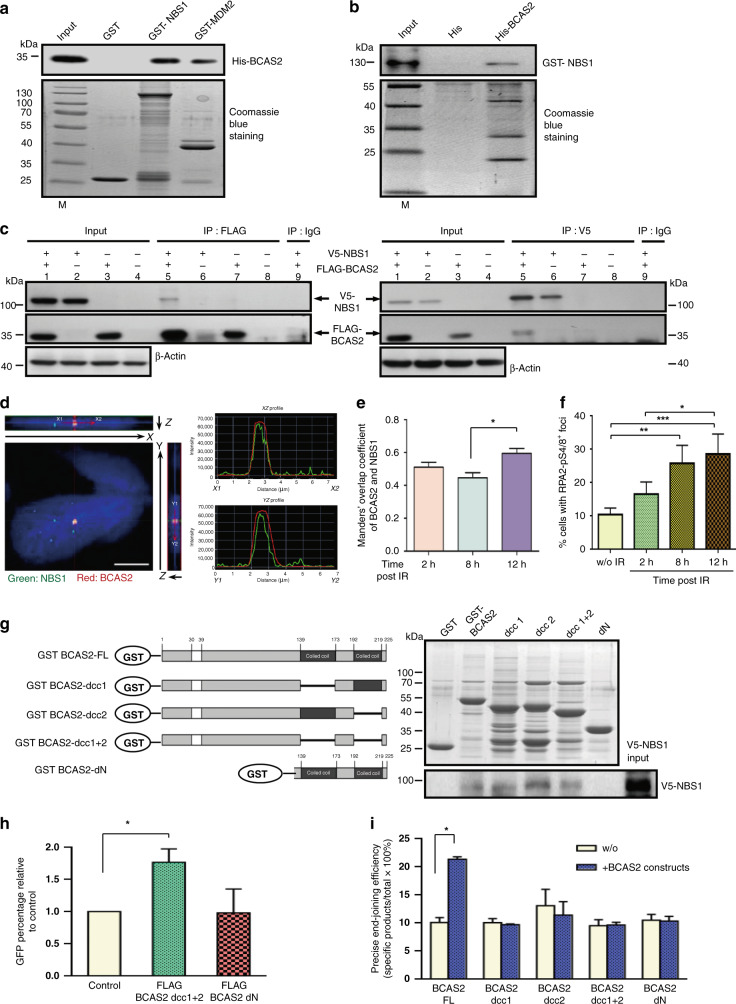
Validation of interaction between BCAS2 and NBS1 and identification of the NBS1-interacting domain in BCAS2. **a**, **b** In vitro pull-down assay. Bacteria-produced GST-NBS1 (**a**) and His-BCAS2 (**b**) proteins were incubated with bead-bound His-BCAS2 and GST-NBS1, respectively. Eluted proteins were analysed by western blotting using anti-His to detect His-BCAS2 or anti-GST antibody to detect GST-NBS1 (upper panels). GST only and GST-MDM2^15^ fragments served as negative and positive controls, respectively. The proper expressions of GST-NBS1 (**a**) and His-BCAS2 (**b**) proteins from bacteria were confirmed by Coomassie blue staining (lower panels). M, protein marker. **c** Reciprocal co-immunoprecipitation assay: HEK 293T cells were transfected with expression vectors for V5-tagged NBS1 or control vector, in addition to either FLAG-tagged BCAS2 expressing plasmids or FLAG vector. Cell extracts were immunoprecipitated (IP) with anti-V5 or anti-FLAG antibodies and immunoblotted using the indicated antibodies. The levels of β-actin were used as loading controls in the input. **d**–**f** Evaluation of the co-localisation status of BCAS2 and NBS1 in U2OS cells. Confocal microscopy analysis (**d**, left panel) showed subcellular co-localisation of BCAS2 (red) and NBS1 (green) foci in a nucleus in Z-stack images (YZ and XZ). Scale bar = 10 μm. The intensity profiles (right panel) along selected lines (X1–X2, and Y1–Y2) demonstrated the overlapping fluorescence signals for BCAS2 and NBS1. **e** Quantitative image analysis of co-localisation was also performed using immunofluorescent images of BCAS2 and NBS1 in U2OS cells taken at 2, 8 and 12 h after ionising radiation (IR) (10 Gy). The mean overall Manders’ overlap coefficient of BCAS2 and NBS1 was calculated from 50 to 60 nuclei per coverslip using the software tool implemented in ImageJ via the JACoP plugin. The histogram shows that the mean overall Manders’ overlap coefficient of NBS1 and BCAS2 at 12 h after IR was significantly (*n* = 3; *P < 0.05; Kruskal–Wallis test with Dunn’s test) greater than that at 8 h. **f** The percentages of cells with RPA2-pS4/8-positive foci during a time course after irradiation or without irradiation. Immunofluorescent microscopy analysis was performed to calculate the percentage of U2OS cells that were positive for phospho-RPA2 (RPA2-pS4/8) foci (the representative images are in Supplementary Fig. S4b) during a time course after IR. The histogram shows that the percentage of cells positive for RPA2-pS4/8 foci at 12 h after IR was significantly higher than that at 2 h or that without irradiation; the percentage at 8 h was also significantly higher than that without irradiation (100 cells per coverslip; *n* = 6; *P < 0.05, **P < 0.01, ***P < 0.001; Kruskal–Wallis test with Dunn’s test). **g**–**i** Identification of the NBS1-binding domain of BCAS2 and its significance in homologous recombination (HR) and non-homologous end joining (NHEJ). **g** Domain mapping showed that the N-terminal domain was required for BCAS2 to interact with NBS1. The lysates of HEK 293T cell transfected with the plasmid expressing V5-NBS1 were incubated with each glutathione resin-bound GST-BCAS2 deletion protein produced by bacteria, and the pulled-down proteins were eluted and subjected to western blotting using anti-V5 antibody to assess the amount of bound NBS1. The proper expression of various deletion proteins of BCAS2, as depicted in a schematic illustration (left panel), from bacteria was confirmed by Coomassie blue staining (right panel). FL full-length, dcc1 deletion of coiled-coil 1 domain, dcc2 deletion of coiled-coil domain 2, dcc1 + 2 deletion of both dcc domains, dN deletion of the N-terminal half. The BCAS2 N-terminus, but not any of the two coiled-coil domains, was essential in enhancing HR activity. **h** Determination of the domain that is important for the effect of BCAS2 on HR. The overexpression of an N-terminal half-deleted BCAS2 (FLAG-BCAS2 dN) had no effect, while the overexpression of a BCAS2 construct with the deletion of both coiled-coil domains (FLAG BCAS2 dcc1 + 2) still exhibited a significant (*P < 0.05; Mann–Whitney *U* test) enhancing effect on HR activity, which is represented by the GFP-positive cell percentage measured by flow cytometry described in Fig. 2e legend, The data were normalised to that of the control (vector only). **i** Full-length BCAS2 was required to enhance the in vivo precise NHEJ activity. Measurement of in vivo precise NHEJ activity is described in detail in “Methods” and in the legend of Fig. 2b. Briefly, the luciferase reporter plasmid was linearised and co-transfected into HEK 293T cells with empty vector control (w/o), the full-length BCAS2 cDNA, or each of the indicated BCAS2 cDNA deletion constructs. The results showed that only the full-length BCAS2 had a significant enhancing effect on precise NHEJ activity. The data are presented as means + SD (*n* = 3; *P < 0.05; Mann–Whitney *U* test).

In comparing cellular proteins pulled down by GST-BCAS2 versus GST using mass spectrometry in our previous published work,^15^ we found that MRE11 was included in the long list of BCAS2-interacting proteins (Supplementary Table 2). However, because NBS1 functions as an adapter protein and contains two forkhead-associated domains that were reported to interact with a coiled-coil domain,^30^ we hypothesised that BCAS2 might interact with the MRE11 complex through NBS1, as BCAS2 also contains coil–coil domains. We then decided to focus on NBS1 first. We produced GST-NBS1 and His-tagged BCAS2 bacterial fusion proteins and performed in vitro reciprocal pull-down assays (Fig. 3a, b). The results showed that His-BCAS2 bound to GST-NBS1 was detected by immunoblotting in the complexes that were pulled down by glutathione-agarose beads (Fig. 3a). Similarly, immunoblotting also identified His-BCAS2-bound GST-NBS1 in the complexes pulled down by the Ni-NTA agarose beads (Fig. 3b). In addition, to demonstrate that BCAS2 and NBS1 interact in vivo, we co-transfected V5-tagged NBS1 and FLAG-BCAS2 plasmids into HEK 293T cells and performed a co-immunoprecipitation assay. The results of immunoblotting indicated that V5-NBS1 was present in the complexes precipitated by an anti-FLAG antibody, and FLAG-BCAS2 was present in the complexes precipitated by an anti-V5 antibody (Fig. 3c). We also conducted confocal microscopy to evaluate the co-localisation status of BCAS2 and NBS1 in U2OS cells 8 h after irradiation. The Z-stack images (Fig. 3d, left panel) and intensity profiles (Fig. 3d, right panel) of the fluorescent signals showed that some, but not all, IR-induced NBS1 and BCAS2 foci overlapped well at the subcellular scale, which suggests that at least some NBS1 and BCAS2 proteins were recruited to the damaged sites and possibly worked in tandem during DDR. We also evaluated the co-localisation of NBS1 and BCAS2 in U2OS cells during a time course by calculating the Manders’ overlap coefficient in double-immunofluorescence microscopy at 2, 8 and 12 h after IR. The results showed that the overall Manders’ overlap coefficient increased significantly at 12 h after IR compared to that at 8 h (Fig. 3e; Supplementary Fig. S4a). To estimate the relative degree of HR after IR during the same time course, we performed immunofluorescence microscopy to detect RPA2-pS4/8, which is a readout for RPA-coated ssDNA. The result demonstrated that U2OS cells at 12 h after IR had a significantly increased percentage of RPA2-pS4/8 positivity compared to those at 2 h or those without IR (Fig. 3f and Supplementary Fig. S4b). In addition, to further map the domains in BCAS2 and NBS1 that are responsible for their mutual interaction, we first produced a series of deletion proteins of GST-BCAS2 (see Fig. 3g, left panel, schematic representation) in bacteria and incubated each of them with lysates from cells transfected with V5-NBS1. The GST pull-down assay showed that all three C-terminal coiled-coil domain-deleted BCAS2 clones, but not the N-terminus-deleted clone were able to bind NBS1 (Fig. 3g, right panel), which suggests that the N-terminus was required for BCAS2 to interact with NBS1. Reciprocally, we incubated GST-BCAS2 with the lysates from the HEK 293T cells transfected with the full-length or each of the three V5-NBS1 deletion clones (Supplementary Fig. S5a, left panel) and performed a GST pull-down assay. The results showed that both the N-terminal and C-terminal V5-NBS1 clones, but not the middle region clone, retained the ability to associate with GST-BCAS2 (Supplementary Fig. S5a, right panel). Congruently, we found that the N-terminus of BCAS2 was required for the upregulation of HR efficiency, and the C-terminus of BCAS2, where coiled-coil domains are located, was dispensable (Fig. 3h). Moreover, we aimed to determine which BCAS2 domain is crucial in enhancing NHEJ by comparing the effect of overexpression of each BCAS2 cDNA deletion construct in HEK 293T cells. The results showed that only the full-length BCAS2 possessed the ability to enhance NHEJ; the loss of either the N-terminus or any coiled-coil domain at the C-terminus negated this ability (Fig. 3i). Together, these results indicated that the BCAS2–NBS1 interaction required the N-terminal domain of BCAS2 and both the N- and C-terminal domains of NBS1. Finally, to determine whether the endogenous BCAS2 and NBS1 proteins interacted with each other, we performed a reciprocal co-immunoprecipitation assay in HEK 293T cells, which confirmed that NBS1 was successfully captured by an anti-BCAS2 antibody, and vice versa (Supplementary Fig. S5b).

### BCAS2 regulates DNA repair by facilitating NBS1 recruitment to DSBs

**Fig. 4:**
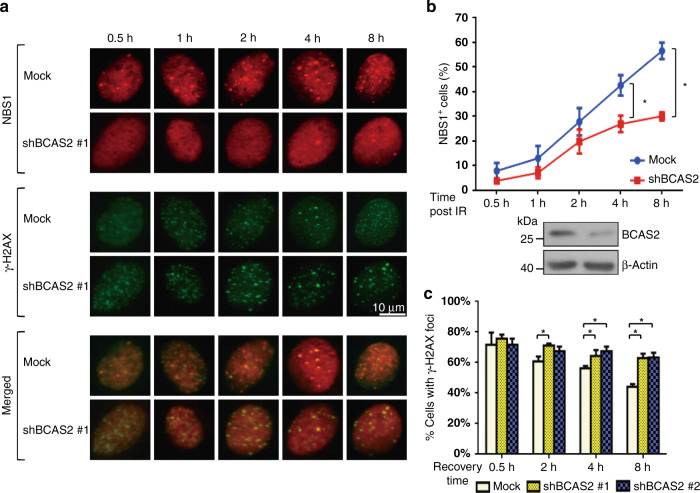
Depletion of BCAS2 reduced NBS1-related DSB repair activities. **a** The BCAS2 shRNA (shBCAS2 #1)- or mock-transfected U2OS cells were exposed to 10 Gy ionising radiation (IR) and then harvested in a series of recovery time points (0.5, 1, 2, 4 and 8 h) for immunofluorescence microscopy to visualise NBS1-positive, γ-H2AX-positive and double-positive foci. Scale bar = 10 μm. **b** Knockdown of BCAS2 significantly decreased the percentage of NBS1 foci-positive cells during a time course after irradiation. U2OS cells containing five or more foci that were positive for both NBS1 and γ-H2AX simultaneously (**a**, lower panels, merged) in the nucleus were defined as NBS1 foci-positive. The percentages of NBS1 foci-positive cells with the knockdown of BCAS2 were significantly lower at 4 h and 8 h after irradiation than those without the BCAS2 knockdown. The knockdown of BCAS2 was confirmed by western blotting (lower panel). Data are presented as means + SD (150 cells per coverslip, *n* = 3; *P < 0.05; Mann–Whitney *U* test). **c** The percentages of γ-H2AX foci-positive cells during a time course after irradiation with or without the knockdown of BCAS2. The depletion of BCAS2 significantly increased the percentage of irradiated cells with γ-H2AX foci at 2, 4 and 8 h. The data are presented as means + SD (50 cells per coverslip; *n* = 3; * *P* < 0.05; Mann–Whitney *U* test).

To explain how BCAS2 affects DNA repair on DDR, we hypothesised that BCAS2 regulates the function of NBS1 during DNA repair processes. The reason is that we showed that BCAS2 interacts with NBS1 both in vitro and in vivo. To prove the hypothesis, we performed a time-course experiment (Fig. 4), in which shBCAS2- or mock-transfected U2OS cells were exposed to IR (10 Gy). Immunofluorescence microscopy (Fig. 4a) was performed to compare the change in percentage of NBS1 foci- or γ-H2AX foci-positive cells at different time points after IR. The results (Fig. 4a, b) showed that compared to the control, the knockdown of BCAS2, resulted in a significant reduction in the percentage of NBS1 foci-positive cells at 4 h (*P* < 0.05) and 8 h (*P* < 0.05) after IR. Moreover, there was a significant increase in the percentage of cells with γ-H2AX foci at 2, 4 and 8 h after IR. Similar results were obtained using the second set of shRNA against BCAS2 (shBCAS2 #2, Fig. 4c and Supplementary Fig. S6). Together, these results suggest that BCAS2 expression facilitates the association of NBS1 with DNA DSBs sites and increases the efficiency of DNA repair.

### BCAS2 expression increases in aggressive human PCa samples, correlates with β-catenin and *NBS1* expression levels and it is associated with shorter survival in PCa patients

**Fig. 5:**
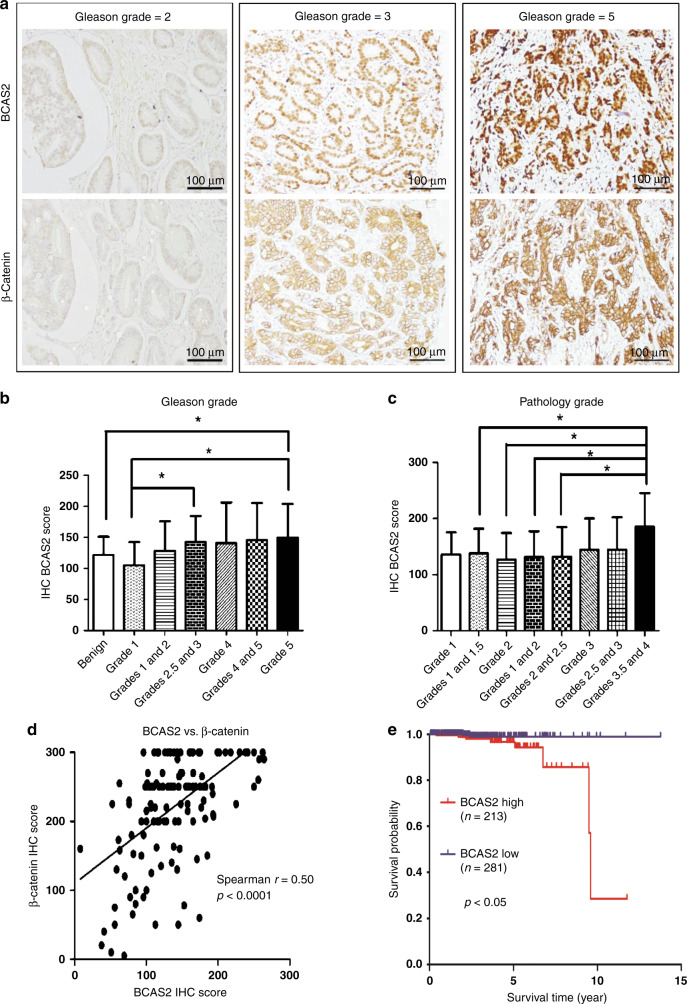
Higher expression of BCAS2 was detected in more aggressive human prostate cancer (PCa) and was associated with the expression of β-catenin. **a** Representative images of IHC staining for BCAS2 and β-catenin in PCa tissues. Lower expression levels in both BCAS2 and β-catenin were found in the same PCa tissue with a Gleason grade equal to 2 (left panels). Note that the upper and lower panels are from sections that are close to each other. Intermediate expression levels of both BCAS2 and β-catenin were observed in the same PCa tissue with a Gleason grade equal to 3 (middle panels). Higher expression levels in both BCAS2 and β-catenin were detected in the same PCa, where the highest Gleason grade equal to 5 (right panels). Brown, positive IHC signals by diaminobenzidine; light blue, haematoxylin counterstain (with decreased staining time to avoid the masking effect of haematoxylin on the IHC signals in the nucleus). Scale bar = 100 μm. **b** The BCAS2 IHC scores significantly differed between benign lesions and Gleason grade 5 tumours, between Gleason grade 1 and grade 5 tumours, and between Gleason grade 1 and grade 2.5–3 tumours (*P < 0.05; Mann–Whitney *U* test). **c** The BCAS2 IHC score of pathology grade 3.5–4 tumours was significantly higher than those of grades 1–1.5, 2, 1–2 or 2–2.5 (*P < 0.05; Mann–Whitney *U* test). **d** The BCAS2 expression level (represented as the IHC score) correlated with β-catenin expression level. The BCAS2 IHC scores exhibited a significant positive correlation with the β-catenin score (Spearman *r* = 0.4979, *P* < 0.0001). PCa (*n* = 118) and benign hyperplasia (*n* = 18) tissue sections were subjected to IHC using anti-BCAS2 or anti-β catenin antibody. We scored the IHC results by multiplying the intensity score of staining (0 ~ +3) and the percentage of the positively stained area. **e** The survival analysis showed that high levels of *BCAS2* mRNA in PCa tissues were significantly correlated with shorter survival in patients with PCa. The high expression level was defined as *BCAS2* mRNA of more than 30.42 fragments per kilobase of exon per million reads (FPKM). The low expression level was defined as equal to or <30.42 FPKM. The survival analysis was performed by the Kaplan–Meier method using GraphPad software and data from The Human Protein Atlas^44^ website (https://www.proteinatlas.org; https://www.proteinatlas.org/ENSG00000116752-BCAS2/pathology/prostate+cancer). RNA sequencing data were reported as the median FPKM and generated by The Cancer Genome Atlas (https://www.genome.gov/Funded-Programs-Projects/Cancer-Genome-Atlas). The log-rank (Mantel–Cox) test was used to evaluate the significance of the difference. Note that PCa patients with a high level of *BCAS2* mRNA (*n* = 213) had significantly shorter (*P* < 0.05) survival than those with a low level of *BCAS2* mRNA (*n* = 281).

To determine whether BCAS2 may serve as a marker of progression and prognosis of human PCa, we performed IHC (Fig. 5a) to stain BCAS2 and β-catenin, which is a potential downstream target of BCAS2, in tissue microarrays that contained benign prostate hyperplasia and PCa tissues. We sought to determine whether there was any correlation between the expression scores and clinical profiles. The results showed that in general, the BCAS2 IHC scores tended to increase in PCa at higher Gleason grades (Fig. 5b) or higher pathology grades (Fig. 5c). Moreover, the IHC score of BCAS2 showed a moderate and positive correlation (Fig. 5d) with that of β-catenin (Spearman *r* = 0.4979, *P* < 0.0001). To complement the results of the IHC study and to evaluate the potential effect of BCAS2 expression on the survival of PCa patients, we performed a survival analysis using the data from The Cancer Genome Atlas. The result showed that compared to those with low *BCAS2* mRNA levels, high levels of *BCAS2* mRNA in PCa tissues were associated with shorter patient survival (*P* < 0.05) (Fig. 5e). Furthermore, in agreement with our results at the molecular level, we found a positive correlation (Spearman *r* = 0.233, *P* < 0.0001) between the mRNA levels of *BCAS2* and *NBS1* in PCa tissues (Supplementary Fig. S7a). Based on the mRNA expression level of *NBS1*, we divided PCa patients with high expressions of *BCAS2* into two groups: one with high levels of *NBS1* and the other with low levels of *NBS1*. The survival analysis showed that only the PCa tissues with high mRNA levels of both *BCAS2* and *NBS1* (Supplementary Fig. S7b, left panel), but not those with high *BCAS2* and low *NBS1* mRNA levels (Supplementary Fig. S7b, right panel), were significantly associated with shorter patient survival (*P* = 0.0067) compared with those PCa tissues with low levels of *BCAS2* mRNA.

## Discussion

Based on our results, we propose that BCAS2 may play a crucial role in DSB repair and have the potential to stimulate the activities of both major DSB repair pathways through interaction with NBS1, which is a key component in the MRN complex. Because cells need to decide whether DSBs are repaired by NHEJ or HR, and NBS1 has been reported to be involved in HR rather than NHEJ,^31^ it seems contradictory that BCAS2, a NBS1-ineracting protein, is able to stimulate the activity of both NHEJ and HR. However, this dual ability also exists in the MRN complex itself because it has an enhancing effect on not only HR but also NHEJ. For instance, it was reported that mammalian cells lacking the component of the MRN complex exhibited reduced NHEJ activity^32,33^ or had impaired ability in executing events requiring NHEJ, such as programmed DNA rearrangement^34^ and the repair of DNA adducts.^35^ This paradox could be explained by the fact that, in addition to the MRN complex, other pathway-specific MRN-interacting proteins are required for cells to make the final decision regarding which pathway to take in DNA repair. The association with different sets of proteins may confer the MRN complex with the ability to enhance the activity of either pathway. Similar explanations may also apply to the situation of BCAS2. We hypothesise that BCAS2 may associate with certain pathway-specific proteins to participate in the decisive step towards only one of the two pathways, depending on which pathway-specific proteins are more abundant or have higher affinity. Alternatively, because BCAS2 lacks any domain with known enzymatic activity, it may not be involved in initiating either pathway, and is only passively recruited by different upstream master complexes to play a structural or adaptor role in subsequent events after cells have selected a pathway. In both scenarios, BCAS2, either directly or indirectly through NBS1, is able to interact with the proteins other than NBS1. Interestingly, our results showed that the BCAS2-mediated enhancement of NHEJ and HR required different protein domains of BCAS2. Both the in vitro domain mapping and the determination of the domain function in HR both showed that only the N-terminus was indispensable for BCAS2 to interact with NBS1 and to enhance HR. In contrast, the in vivo NHEJ assay showed that only the full-length BACS2 possessed the ability to stimulate NHEJ activity. Together, these results suggest that BCAS2 might use different domains to associate with non-NBS1 factors that may contribute to the enhancement of HR or NHEJ. This possibility is consistent with our hypothesis that through interaction with different pathway-specific factors, BCAS2 may acquire the ability to stimulate the activity of either DSB repair pathway, depending on which pathway-specific factors are predominantly present in the cells at that particular moment.

In this study, we demonstrated that there is a direct interaction between BCAS2 and NBS1, and it is likely that BCAS2 participates in DNA repair through NBS1. However, it remains to be determined exactly how BCAS2 regulates the activity of NBS1 or the MRN complex. We did not observe that BCAS2 increased the phosphorylation of NBS1 on Phos-tag acrylamide gel (data not shown). It is possible that BCAS2 regulates NBS1 by other mechanisms. For example, CtIP endonuclease interacts directly with NBS1, forming a CtIP–MRN complex and translocating to DNA break sites through the DSB sensor ability of MRN.^36^ The CtIP–MRN complex activates ATM kinase, which is followed by executing DSB resection to produce RPA-coated ssDNA, leading to cell-cycle checkpoint activation and HR repair.^36^ It is possible that BCAS2 facilitates the transition of MRN activities, from DSB sensing to engagement in the DSB repair processing, although BCAS2 does not have the Sae2-like domain of CtIP, which encodes a 5′ to 3′ endonuclease to remove small nucleotides from the ends. Another possible mechanism might be similar to that of ATMIN, which functions as an activator of ATM in response to chromatin changes. In the canonical IR-induced ATM pathway, the ATMIN protein competes for ATM to interact with NBS1, and it may act as a repair-associated scaffold in regulating DSB repair through controlling the function of ATM.^37^ Whether BCAS2 interacts with CtIP, ATMIN, ATM or other NBS1 regulators or effectors warrants further study.

In addition to binding to NBS1, BCAS2 may regulate the activity of the MRN complex by other mechanisms. For example, we previously identified MRE11 in GST-BCAS2 pull-down samples that were analysed by liquid chromatography-mass spectrometry (Supplementary Table S2).^15^ We also detected poly ADP-ribose polymerase-1 in the proteins co-immunoprecipitated by an anti-BCAS2 antibody (data not shown). Poly ADP-ribose polymerase-1 is a nuclear enzyme required for the accumulation of MRE11 and NBS1 at DSB sites.^38^ In addition, it is possible that BCAS2 may affect DSB repair through interacting with other essential factors and thus act as a recruiter or scaffolding protein for other DSB factors. A well-known example is p29, which has a molecular weight similar to BCAS2, and it has been reported to interact with and increase the phosphorylation of Chk1/Chk2 proteins, resulting in an elevated amount of chromatin-bound MRN/ATRIP.^39^ BCAS2 has also been observed to interact with RPA, activate ATR, and facilitate the phosphorylation of Chk1 and RPA2;^20^ the latter is known to interact with and regulate the MRN complex.

It is also possible that BCAS2 is involved in DNA repair other than HR and NHEJ. Recent studies have revealed that the MRN complex is involved in microhomology-mediated end joining (MMEJ), a major pathway for Ku-independent alternative NHEJ, which contributes to chromosomal translocations and telomere fusions.^40^ MMEJ uses the 5–25 base-pair microhomologous sequences aligned at the adjacent broken ends before joining, thereby resulting in deletions of variable lengths. MRN has been reported to facilitate classical and alternative NHEJ pathways by stabilising distant breaks and processing DNA termini.^41^ In this study, we showed that BCAS2 is able to interact with NBS1, which is a component of MRN. Hence, it would be interesting to determine whether BCAS2 participates in MMEJ variant pathways.^42^

Our IHC results in human PCa tissues showed that, in general, BCAS2 protein was expressed at significantly higher levels in PCa with higher Gleason grades or pathology grades, and that BCAS2 expression levels were positively correlated with β-catenin levels. This finding is reasonable because β-catenin is a pivotal effector of the Wnt signalling pathway, and it is closely related to progression and metastasis in many cancers, including PCa. In addition, we demonstrated that BCAS2 maintains the proper dendrite growth in the mouse brain by upregulating the expression of the β-catenin gene.^43^ It is likely that BCAS2 is also an upstream regulator of β-catenin in PCa. Thus, on the one hand, BCAS2 is capable of enhancing the efficiency of DNA repair. On the other hand, BCAS2 may activate β-catenin and the oncogenes downstream. The possible synergistic effect of both mechanisms may explain the role of BCAS2 in promoting carcinogenesis and/or tumour progression.

In summary, using in vitro and in vivo models, we demonstrate that BCAS2 enhances the efficiency of DNA repair after IR-induced DNA damage. Our results also reveal that BCAS2 promotes the efficiency of both HR and NHEJ, possibly by interacting with and regulating NBS1. Moreover, we demonstrate that the expression of BCAS2 protein significantly increases in human PCa with highly malignant potential. We also show that the overexpression of BCAS2, especially when NBS1 is also overexpressed, is significantly correlated with the probability of shorter survival. Thus, our results may provide a basis for developing a novel strategy for treating human PCa by the genetic and/or pharmacological targeting of BCAS2.

## Supplementary information

All supplementary data in one file

## Data Availability

The datasets used and/or analysed during this study are available from the corresponding author on reasonable request.

## References

[1] Basu, A. K. DNA damage, mutagenesis and cancer. *Int. J. Mol. Sci*. **19**, 970 (2018). 10.3390/ijms19040970 PMC5979367 29570697

[2] Rupnik A, Lowndes NF, Grenon M (2010). MRN and the race to the break. Chromosoma.

[3] Panier S, Boulton SJ (2014). Double-strand break repair: 53BP1 comes into focus. Nat. Rev. Mol. Cell Biol..

[4] Vignard J, Mirey G, Salles B (2013). Ionizing-radiation induced DNA double-strand breaks: a direct and indirect lighting up. Radiother. Oncol..

[5] Burma S, Chen BP, Chen DJ (2006). Role of non-homologous end joining (NHEJ) in maintaining genomic integrity. DNA Repair.

[6] Rein K, Stracker TH (2014). The MRE11 complex: an important source of stress relief. Exp. Cell Res..

[7] Rupnik A, Grenon M, Lowndes N (2008). The MRN complex. Curr. Biol..

[8] Jungmichel S, Stucki M (2010). MDC1: the art of keeping things in focus. Chromosoma.

[9] Lamarche BJ, Orazio NI, Weitzman MD (2010). The MRN complex in double-strand break repair and telomere maintenance. FEBS Lett..

[10] Yuichiro SaitoHF, Junya Kobayashi (2013). Role of NBS1 in DNA damage response and its relationship with cancer development. Transl. Cancer Res..

[11] Lu X, Legerski RJ (2007). The Prp19/Pso4 core complex undergoes ubiquitylation and structural alterations in response to DNA damage. Biochem. Biophys. Res. Commun..

[12] Henriques JA, Vicente EJ, Leandro, da Silva KV, Schenberg AC (1989). PSO4: a novel gene involved in error-prone repair in Saccharomyces cerevisiae. Mutat. Res..

[13] Grey M, Dusterhoft A, Henriques JA, Brendel M (1996). Allelism of PSO4 and PRP19 links pre-mRNA processing with recombination and error-prone DNA repair in Saccharomyces cerevisiae. Nucleic Acids Res..

[14] Kleinridders A, Pogoda HM, Irlenbusch S, Smyth N, Koncz C, Hammerschmidt M (2009). PLRG1 is an essential regulator of cell proliferation and apoptosis during vertebrate development and tissue homeostasis. Mol. Cell Biol..

[15] Kuo PC, Tsao YP, Chang HW, Chen PH, Huang CW, Lin ST (2009). Breast cancer amplified sequence 2, a novel negative regulator of the p53 tumor suppressor. Cancer Res..

[16] Kuo PC, Huang CW, Lee CI, Chang HW, Hsieh SW, Chung YP (2015). BCAS2 promotes prostate cancer cells proliferation by enhancing AR mRNA transcription and protein stability. Br. J. Cancer.

[17] Chen PH, Lee CI, Weng YT, Tarn WY, Tsao YP, Kuo PC (2013). BCAS2 is essential for Drosophila viability and functions in pre-mRNA splicing. RNA.

[18] Chou MH, Hsieh YC, Huang CW, Chen PH, Chan SP, Tsao YP (2015). BCAS2 regulates delta-notch signaling activity through delta pre-mRNA splicing in Drosophila wing development. PLoS ONE.

[19] Liao S, Du R, Wang L, Qu Z, Cui X, Li C (2015). BCAS2 interacts with HSF4 and negatively regulates its protein stability via ubiquitination. Int. J. Biochem. Cell Biol..

[20] Wan L, Huang J (2014). The PSO4 protein complex associates with replication protein A (RPA) and modulates the activation of ataxia telangiectasia-mutated and Rad3-related (ATR). J. Biol. Chem..

[21] Xu Q, Wang F, Xiang Y, Zhang X, Zhao ZA, Gao Z (2015). Maternal BCAS2 protects genomic integrity in mouse early embryonic development. Development.

[22] Bau DT, Fu YP, Chen ST, Cheng TC, Yu JC, Wu PE (2004). Breast cancer risk and the DNA double-strand break end-joining capacity of nonhomologous end-joining genes are affected by BRCA1. Cancer Res..

[23] Chu YL, Wu X, Xu Y, Her C (2013). MutS homologue hMSH4: interaction with eIF3f and a role in NHEJ-mediated DSB repair. Mol. Cancer.

[24] Nakanishi K, Cavallo F, Brunet E, Jasin M (2011). Homologous recombination assay for interstrand cross-link repair. Methods Mol. Biol..

[25] Tehranian N, Sepehri H, Mehdipour P, Biramijamal F, Hossein-Nezhad A, Sarrafnejad A (2012). Combination effect of PectaSol and doxorubicin on viability, cell cycle arrest and apoptosis in DU-145 and LNCaP prostate cancer cell lines. Cell Biol. Int..

[26] Namdar M, Perez G, Ngo L, Marks PA (2010). Selective inhibition of histone deacetylase 6 (HDAC6) induces DNA damage and sensitizes transformed cells to anticancer agents. Proc. Natl Acad. Sci. USA.

[27] Madigan JP, Chotkowski HL, Glaser RL (2002). DNA double-strand break-induced phosphorylation of Drosophila histone variant H2Av helps prevent radiation-induced apoptosis. Nucleic Acids Res..

[28] Bau DT, Mau YC, Ding SL, Wu PE, Shen CY (2007). DNA double-strand break repair capacity and risk of breast cancer. Carcinogenesis.

[29] Pierce AJ, Johnson RD, Thompson LH, Jasin M (1999). XRCC3 promotes homology-directed repair of DNA damage in mammalian cells. Genes Dev..

[30] Lee JR, Shin H, Choi J, Ko J, Kim S, Lee HW (2004). An intramolecular interaction between the FHA domain and a coiled coil negatively regulates the kinesin motor KIF1A. EMBO J..

[31] Bennardo N, Gunn A, Cheng A, Hasty P, Stark JM (2009). Limiting the persistence of a chromosome break diminishes its mutagenic potential. PLoS Genet..

[32] Huang J, Dynan WS (2002). Reconstitution of the mammalian DNA double-strand break end-joining reaction reveals a requirement for an Mre11/Rad50/NBS1-containing fraction. Nucleic Acids Res..

[33] Rass E, Grabarz A, Plo I, Gautier J, Bertrand P, Lopez BS (2009). Role of Mre11 in chromosomal nonhomologous end joining in mammalian cells. Nat. Struct. Mol. Biol..

[34] Dinkelmann M, Spehalski E, Stoneham T, Buis J, Wu Y, Sekiguchi JM (2009). Multiple functions of MRN in end-joining pathways during isotype class switching. Nat. Struct. Mol. Biol..

[35] Quennet V, Beucher A, Barton O, Takeda S, Lobrich M (2011). CtIP and MRN promote non-homologous end-joining of etoposide-induced DNA double-strand breaks in G1. Nucleic Acids Res..

[36] You Z, Shi LZ, Zhu Q, Wu P, Zhang YW, Basilio A (2009). CtIP links DNA double-strand break sensing to resection. Mol. Cell.

[37] Zhang T, Penicud K, Bruhn C, Loizou JI, Kanu N, Wang ZQ (2012). Competition between NBS1 and ATMIN controls ATM signaling pathway choice. Cell Rep..

[38] Haince JF, McDonald D, Rodrigue A, Dery U, Masson JY, Hendzel MJ (2008). PARP1-dependent kinetics of recruitment of MRE11 and NBS1 proteins to multiple DNA damage sites. J. Biol. Chem..

[39] Chu PC, Wang TY, Lu YT, Chou CK, Yang YC, Chang MS (2009). Involvement of p29 in DNA damage responses and Fanconi anemia pathway. Carcinogenesis.

[40] Truong LN, Li Y, Shi LZ, Hwang PY, He J, Wang H (2013). Microhomology-mediated End Joining and Homologous Recombination share the initial end resection step to repair DNA double-strand breaks in mammalian cells. Proc. Natl Acad. Sci. USA.

[41] Xie A, Kwok A, Scully R (2009). Role of mammalian Mre11 in classical and alternative nonhomologous end joining. Nat. Struct. Mol. Biol..

[42] Kostyrko K, Mermod N (2016). Assays for DNA double-strand break repair by microhomology-based end-joining repair mechanisms. Nucleic Acids Res..

[43] Huang CW, Chen YW, Lin YR, Chen PH, Chou MH, Lee LJ (2016). Conditional knockout of breast carcinoma amplified sequence 2 (BCAS2) in mouse forebrain causes dendritic malformation via beta-catenin. Sci. Rep..

[44] Uhlen M, Fagerberg L, Hallstrom BM, Lindskog C, Oksvold P, Mardinoglu A (2015). Proteomics. Tissue-based map of the human proteome. Science.

