# Supplementary material for: BCAS2, a protein enriched in advanced prostate cancer, interacts with NBS1 to enhance DNA double-strand break repair
Source: Br J Cancer. 2020 Sep 23;123(12):1796–807. doi: 10.1038/s41416-020-01086-y (PMC7723048; doi:10.1038/s41416-020-01086-y)
Supplement: Supplementary file 1 — All supplementary data in one file [file 41416_2020_1086_MOESM1_ESM.pdf]

SUPPLEMENTARY MATERIALS AND METHODS

Lentiviral particle preparation and infection

Small hairpin RNAs against human BCAS2 (shBCAS2) were constructed in the pLKO.1-puro lentiviral vector. The target sequences are shBCAS2 #1: 5’-AGACTGGCTGCTCGACAAC-3’ and shBCAS2 #2: 5’-GATTGAACACGCACAGAAG-3’. The pLKO.1-puro vector and the control scrambled shRNA vector were obtained from the National RNAi Core Facility of Academia Sinica, Taiwan. The production of lentivirus particles was as previously described.[1] Cells for infection were seeded at 70% confluence. One day after seeding, lentiviral infection was performed by adding 33% (v/v) of lentivirus-containing medium and polybrene (10 μg/mL) to the regular culture medium. The stable pools of infected cells were selected using 2 μg/ml puromycin for 4–7 days before using the cells in experiments. For the overexpression of BCAS2, the cDNA of BCAS2 was cloned into a lentivirus-based vector named pSIN-MCS, which was used for transfecting HEK 293T cells to produce viruses. After infecting the cells with lentivirus for 4 days, we collected viable cells without selection for the subsequent assays in this study.

Quantitative real-time PCR

RNA was isolated with TRIZOL (Invitrogen), and the first strand cDNA was synthesized using 1 μg of RNA and SuperScript III Reverse Transcriptase (Invitrogen). Quantitative real-time PCR (Q-RT-PCR) was performed using specific primers to BCAS2 (forward: 5’-CCTGACAGCCCCGGATTATTCT-3’, reverse: 5’-GGGGCTGGAAGCTCATATCGTT-3’) and Maxima SYBR Green/ROX qPCR

Master Mix (Thermo). BCAS2 RNA levels were normalized to the level of β-actin (Origene).

Cell cycle analysis

HEK 293T cells transfected with the indicated lentiviral shBCAS2 plasmids were trypsinized into single cells, stained with propidium iodide (50 μg/mL), and subjected to cell cycle analysis using a flow cytometer (BD FACSCalibur).

Western blotting

Cells were lysed with RIPA buffer (50 mM pH 8.0 Tris-HCl, 150 mM NaCl, 1% NP-40, 0.5% sodium deoxycholate and protease inhibitors) and the supernatant was collected after centrifugation. Cell lysates were separated by sodium dodecyl sulfate (SDS)-polyacrylamide gel electrophoresis, and then transferred to polyvinylidene difluoride membranes (Millipore), blotted with the indicated primary and horseradish peroxidase-conjugated secondary antibodies, and visualised using an enhanced chemiluminescence western blotting detection system (GE Healthcare). All antibodies used are listed in Supplementary Table 1.

Immunofluorescence microscopy

U2OS cells were seeded on glass coverslips before harvesting. They were fixed in 4% paraformaldehyde and permeabilized with 0.1% Triton X-100 solutions, followed by blocking in 5% blocking solution. The cells were then incubated in the properly diluted antibodies (Supplementary Table 1) in a humidified chamber overnight at 4°C. After incubation with the secondary antibodies conjugated to fluorophores, the cells were stained with 4',6-diamidino-2-phenylindole (DAPI), mounted with mounting medium (Dako, Glostrup, Denmark) and observed with a fluorescence microscope. *Drosophila* wing imaginal discs were dissected from the wandering third instar larvae of specific BCAS2-expressing genotypes. Samples were processed according to the method described previously.[2] Briefly, they were incubated with anti-cleaved caspase 3 antibody overnight at 4°C, followed by the fluorescent secondary antibody, and then observed by confocal microscopy.

Colocalisation study and confocal microscopy

Double immunofluorescence microscopy was performed as described above to detect BCAS2 and NBS1 in U2OS cells and fluorescent images were captured with a CCD camera (LAS-4000, Fujifilm, Japan). Composite images were created by superposition of fluorescence images and subjected to colocalisation analysis. The overall Manders' overlap coefficient of BCAS2 and NBS1 at each time point was

calculated from 50–60 cell nuclei per 20 high-power field images per coverslip (original magnification: 1000 ×) using a colocalisation tool implemented in the ImageJ via the JACoP plugin (https://imagej.nih.gov/ij/plugins/track/jacop.html).[3] The colocalisation study using confocal microscopy was performed with a confocal microscope (TCS SP5, Leica, German). Z-stack images (XZ and YZ) were generated by combining a series of images (total 15 images) with incremental focuses. The intensity profile along the line through the region of interest was generated using the Zen microscope software (Zeiss).

Recombinant protein purification and *in vitro* pull-down assay

The glutathione-S-transferase **(**GST) fusion proteins were expressed in *E. coli* BL21 (DE3) and Rosetta cells (Novagen). Further purifications were performed using glutathione-Sepharose 4B beads (GE Healthcare), according to the manufacturer's protocols. His-tagged proteins were purified using Ni–NTA agarose beads (Qiagen). We obtained the nuclear extract from HEK 293T cells using a nuclear extraction kit (Abcam) and following the manufacturer's instructions. Nuclear extracts and His-tagged proteins were incubated with the GST-fused protein beads. The interacting proteins were eluted and resolved by SDS- polyacrylamide gel electrophoresis and detected by western blotting with the indicated antibodies (Supplementary Table 1).

Supplementary table 1. The list of antibodies used in this study

Primary Ab	Manufacturer	Dilution	Secondary Ab	Manufacturer	Method
Rabbit anti-BCAS2	Bethyl	1:10000	Donkey anti rabbit IgG-HRP	Merck	WB
	Proteintech	1:10000	Donkey anti rabbit IgG-HRP	Merck	WB
		1:50	Goat anti rabbit IgG-Cyanine Cy™3	Jackson ImmunoResearch	IF
		1:100	Donkey anti rabbit IgG-HRP	Merck	IP
		1:500	polymer-HRP	BioGenex	IHC
Mouse anti γ-H_2AX	Merck	1:2000	Goat anti mouse IgG-HRP	Merck	WB
		1:500	Goat anti mouse IgG-Alex Fluor® 488	Jackson ImmunoResearch	IF
Rabbit anti H_2AX	GeneTex	1:10000	Donkey anti rabbit IgG-HRP	Merck	WB
Mouse anti β-actin	Merck	1:10000	Goat anti mouse IgG-HRP	Merck	WB
Rabbit anti Caspase-3	Abcam	1:500	Goat anti rabbit IgG-Cyanine Cy™3	Jackson ImmunoResearch	IF
Mouse anti FLAG	Merck	1:10000	Goat anti mouse IgG-HRP	Merck	WB
		1:500	Goat anti mouse IgG	Thermo	IP
Rabbit anti GST	Santa Cruz	1:2500	Donkey anti rabbit IgG-HRP	Merck	WB
Mouse anti His	Santa Cruz	1:5000	Goat anti mouse IgG-HRP	Merck	WB
Mouse anti V5	Thermo	1:3000	Goat anti mouse IgG-HRP	Merck	WB

		1:250	Goat anti mouse IgG	Thermo	IP
Mouse anti NBS1	BD	1:100	Goat anti mouse IgG-Alex Fluor® 488	Jackson ImmunoResearch	IF
Rabbit anti NBS1	Novus	1:50	Goat anti rabbit IgG-Cyanine Cy™3	Jackson ImmunoResearch	IF
Rabbit anti β-catenin	Abcam	1:100	polymer-HRP	BioGenex	IHC
Rabbit anti phospho RPA32 (S4/S8)	Bethyl	1:500	Goat anti rabbit IgG-Cyanine Cy™3	Jackson ImmunoResearch	IF

WB: western blotting; IF: immunofluorescence; IHC: immunohistochemistry, IP: immunoprecipitation.

Supplementary table 2. The list of BCAS2-interacting proteins. BCAS2 interaction partners were identified and characterised using glutathione S-transferase (GST) pull-down assay followed by mass spectrometry analysis using the extracts of MCF-7 cells.[4] The list is available through the following web link: https://drive.google.com/file/d/115_ZOcRYEF1OQsfJIe6ookyvVNmIGU9u/view?usp=sharing.

SUPPLEMENTARY FIGURE LEGENDS

Fig. S1. The efficiency of BCAS2 knockdown and the effect of BCAS2 expression on the survival of PCa cells treated with DSBs-causing drugs. **a** The efficiency of BCAS2 knockdown was assayed by Q-RT-PCR. To examine the efficiency of BCAS2 knockdown at the mRNA level, LNCaP cells (left panel) and PC-3 cells (right panel) were infected with lentiviruses carrying the shRNA against BCAS2 (shBCAS2 #1 and #2) or a scrambled shRNA (shscramble), or they were mock infected. Cells were selected using puromycin for 4 days and the stable pools were collected for experiments. The cells were exposed to 10Gy γ-radiation, followed by an 8h recovery period. RNA was extracted from irradiated and non-irradiated cells with TRIzol reagent, followed by Q-RT-PCR using the SYBR® Green qPCR system and specific primer pairs. The expression level of *BCAS2* was normalised to that of internal control *ACTB* gene and to the control groups, and presented as means plus SD (n = 3; knockdown *vs* mock or shscramble, *$p < 0.05$; Mann-Whitney U test). The experiments in Fig. 1a-c, including Q-RT-PCR, were repeated for four times and representative results are shown. **b, c.** BCAS2 expression in human PCa cells decreased the cell death caused by chemotherapeutic drugs causing double strand DNA breaks. LNCaP cells (left panels) and PC-3 cells (right panels) were infected with lentiviruses expressing the shRNA for BCAS2 (shBCAS2 #1) (**b**), the FLAG-

BCAS2 cDNA (**c**), or mock infected four days before drug treatment. LNCaP cells were then treated with 100 nM doxorubicin (Dox.), 25 μM etoposide (Etop.), or the solvent (DMSO) for 3 days. The selection of the dosage for both drugs was based on previously published literature.[5,6] Trypan blue exclusion assays were then performed at the end of day 3 using 0.4% trypan blue dye to count the number of surviving cells. In either doxorubicin- or etoposide-treated LNCaP cells at day 3, BCAS2 depletion caused a significantly decreased number of viable cells compared to the mock group (n = 3; $*p < 0.05$). Similarly, BCAS2-depleted PC-3 cells treated with doxorubicin (500 nM) or etoposide (25 μM) for 3 days exhibited significantly decreased viability compared to the untreated controls at day 3 (n = 3; $*p < 0.05$; Mann-Whitney U test). **c** By contrast, BCAS2-overexpressing LNCaP cells (left panel) had a significantly increased number of viable cells after treatment with a low dose of doxorubicin (2.5 nM) or etoposide (75 nM) for 3 days compared to the control groups (at day 3; n = 3; $*p < 0.05$; Mann-Whitney U test). Lower dosages of both drugs were used here to better observe the positive effect of BCAS2 on cell survival. Similarly, PC-3 cells (right panels) with overexpression of BCAS2 showed a significantly higher number of viable cells after treatment with doxorubicin (17.5 nM) or etoposide (200 nM) for 3 days (at day 3; n = 3; $*p < 0.05$; Mann-Whitney U test). All data are presented as means ± SD.

Fig. S2 BCAS2 mitigated the severity of irradiation-induced and p53-independent apoptosis. In the *Drosophila* third instar larvae with the p53-null phenotype, we used *ms1096* to drive $dBCAS2^{dsRNA}$ specifically in the wing discs (cross-shape pattern), so that the expression of *dBCAS2* was selectively knocked down in the wing discs (*ms1096*> $dBCAS2^{dsRNA}$) compared to the wild type (*ms1096*> +). The larvae were irradiated (10 Gy) or not irradiated (0 Gy) IR, followed by a recovery period of 4–6 h at room temperature. The wing discs were then dissected and subjected to immunofluorescence microscopy for cleaved caspase-3 (C3, red), which revealed the location of apoptotic cells in imaginal wing discs caused by irradiation, and for Delta (green), which is a product of a downstream target gene of BCAS2[7] and acts as a proxy for the measurement of the BCAS2 activity. The histogram (right panel) shows that the number of apoptotic cells was significantly increased in the BCAS2-depleted wing discs compared to the control. Data are presented as means plus SD (n = 3; *$p <$ 0.05; Mann-Whitney U test).

Fig. S3. Downregulation of BCAS2 repressed the efficiency of repair of double strand DNA breaks (DSBs) involving RPA2-pS4/8 recruitment. **a, b** HEK 293T cells (**a**) and PC-3 cells (**b**) infected with the lentiviral shRNAs (shBCAS2 #1, #2) or control

shRNA (shscramble) were selected with puromycin. The stable pools of cells were then collected. Cells were then harvested along a time course after IR (10 Gy) or without IR and subjected to double immunofluorescence microscopy to detect γ-H2AX and RPA2-pS4/8 foci. The percentage of γ-H2AX and RPA2-pS4/8 foci double-positive cells in γ-H2AX foci-positive cells (the number of cells with both γ-H2AX and RPA2-pS4/8 foci divided by the number of total cells × 100%) was calculated by counting 50 cells per coverslip (n = 3) at each time point. The original images at 8 h after IR are selected and shown (lower panels). The histograms (upper panels) show that knockdown of BCAS2 significantly repressed the percentage of DSB-positive cells (with γ-H2AX foci) that recruited RPA2-pS4/8 at almost all time points of examination in both cell lines. The effect was more obvious at later time points than at earlier time points in HEK293T cells. Data are presented as means plus SD (n = 3; $*p < 0.05$; Mann-Whitney U test). Scale bar = 10 μm.

Fig. S4. The overlapping status of BCAS2 and NBS1 foci and the number of cells with RPA2-pS4/8 foci in a time course after irradiation (IR). **a** The status of overlap between BCAS2 and NBS1 foci after IR. U2OS cells were irradiated (10 Gy) and harvested at indicated time points after IR, followed by double immunofluorescence microscopy to evaluate the colocalisation or overlapping status of BCAS2 (red) and

NBS1 (green) foci. A representative magnified image containing a single nucleus (the rightmost column) is shown for each time point. The overall Manders' overlapping coefficient was calculated using ImageJ and the result of statistical analysis is shown in Fig. 3e. Scale bar = 10 μm. **b** The number of cells with RPA2-pS4/8 foci in a time course after IR or without IR. U2OS cells were harvested at different time points after IR (10 Gy) and subjected to immunofluorescence microscopy to detect RPA2-pS4/8 foci. Representative images from each time point are shown. The percentage of cells positive for RPA2-pS4/8 foci was calculated by counting 100 cells per coverslip (n = 3) for each time point, and the results are shown in the histogram in Fig. 3f. Scale bar = 10 μm.

Fig. S5. Mapping for BCAS2-interacting domain in NBS1, and interaction between endogenous BCAS2 and NBS1. **a** Identification of the BCAS2-binding domain of NBS1. The lysates of HEK 293T cells that expressed each different deleted V5-NBS1 construct were incubated with the resin-bound full-length GST-BCAS2; bound proteins were eluted from the glutathione resins and immunoblotted using an anti-V5 antibody. The left panel is a schematic representation of various deletion and epitope-tagged constructs of NBS1 protein (C′, C-terminus half only; Mi, the middle part only; N′, N-terminal half only; and FL, full length). The immunoblotting result (lower panel)

suggested that both the N- and C-terminal regions of NBS1, but not the middle region, participated in the interaction between BCAS2 and NBS1. **b** The association of endogenous BCAS2 and NBS1 *in vivo*. HEK 293T cell lysates were obtained with RIPA lysis buffer without detergent, and then sonicated on ice to lyse the nuclei and release nuclear proteins. Endogenous BCAS2 protein was immunoprecipitated with an anti-BCAS2 antibody (2 μg) from the lysates (2mg). The product after immunoprecipitation was subjected to SDS-PAGE and immunoblotting using an anti-NBS1 antibody and another anti-BCAS2 antibody (left panel). Mouse IgG was used as a control. IP: immunoprecipitation; IB: immunoblotting. Reciprocally (right panel), endogenous NBS1 protein was immunoprecipitated from cell lysates by an anti-NBS1 antibody, followed by SDS-PAGE and immunoblotting to capture endogenous BCAS2. The faint IB signal (~ 25 kDa) in the lane of IgG might be the residual light chain of IgG.

Fig. S6 Knockdown of BCAS2 reduced DNA repair-associated NBS1 recruitment to DSBs. **a** BCAS2 knockdown decreased NBS1 recruitment to DSB sites caused by irradiation. U2OS cells were transfected with a second set of shBCAS2 plasmid (shBCAS2 #2). They were irradiated at a dosage of 10 Gy and collected after 8 h of recovery time and subjected to immunofluorescence microscopy using anti-NBS1 and

anti-γ-H2AX antibodies. The presence of five or more NBS1-foci that were colocalised with γ-H2AX-positive foci per cell was defined as NBS1 foci-positive. Mock-transfected cells served as control. Scale bar = 10 μm. **b** The histogram shows the percentages of NBS1-positive cells in all γ-H2AX-positive cells under different conditions. We counted 150 cells from each of the three independent experiments (*$p < 0.05$; Mann-Whitney U test). **c** Knockdown of BCAS2 by shBCAS2 #2 was confirmed by western blotting.

Fig. S7 Positive correlation of mRNA levels between *BCAS2* and *NBS1* and association of high RNA levels of both *BCAS2* and *NBS1* with shorter survival in patients with PCa. **a** The *BCAS2* mRNA level (represented as FPKM) exhibited a significant correlation with the *NBS1* mRNA level (n = 494, Spearman r = 0.2331, $p < 0.0001$). Correlation analysis was performed using the RNA sequencing data from The Human Protein Atlas website (https://www.proteinatlas.org). **b** High mRNA levels of both *BCAS2* and *NBS1* in PCa tissues were associated with shorter survival in patients with PCa. The cutoff value of "high" level of *BCAS2* mRNA was defined as 30.42 FPKM, while the "low" level of *BCAS2* was defined as equal or less than 30.42 FPKM. Similarly, the cutoff value of "high" level of *NBS1* mRNA was defined as 10.04 FPKM. Survival analysis was performed by the Kaplan-Meier method using

GraphPad software and the data from The Human Protein Atlas website (https://www.proteinatlas.org), which provides the PCa RNA sequencing data reported as the median FPKM and generated by The Cancer Genome Atlas. The log-rank (Mantel-Cox) test was used to evaluate the significance of difference. Note that patients with PCa with high mRNA levels of both *BCAS2* and *NBS1* (left panel, n = 110), but not those with high *BCAS2* and low *NBS1* mRNA levels (right panel, n = 103), had significantly (p = 0.0067) shorter survival than patients with low BCAS2 mRNA level (n = 281) in the PCa tissues.

REFERENCES

1. Ko, C. J., Huang, C. C., Lin, H. Y., Juan, C. P., Lan, S. W., Shyu, H. Y. et al. Androgen-Induced TMPRSS2 Activates Matriptase and Promotes Extracellular Matrix Degradation, Prostate Cancer Cell Invasion, Tumor Growth, and Metastasis. *Cancer Res.* **75**, 2949-2960 (2015).
2. Chen, P. H., Lee, C. I., Weng, Y. T., Tarn, W. Y., Tsao, Y. P., Kuo, P. C. et al. BCAS2 is essential for Drosophila viability and functions in pre-mRNA splicing. *RNA* **19**, 208-218 (2013).
3. Bolte, S., Cordelieres, F. P. A guided tour into subcellular colocalization analysis in light microscopy. *J. Microsc.* **224**, 213-232 (2006).
4. Kuo, P. C., Tsao, Y. P., Chang, H. W., Chen, P. H., Huang, C. W., Lin, S. T. et al. Breast cancer amplified sequence 2, a novel negative regulator of the p53 tumor suppressor. *Cancer Res.* **69**, 8877-8885 (2009).
5. Namdar, M., Perez, G., Ngo, L., Marks, P. A. Selective inhibition of histone deacetylase 6 (HDAC6) induces DNA damage and sensitizes transformed cells to anticancer agents. *Proc. Natl. Acad. Sci. U S A* **107**, 20003-20008 (2010).
6. Tehranian, N., Sepehri, H., Mehdipour, P., Biramijamal, F., Hossein-Nezhad, A.,

Sarrafnejad, A. et al. Combination effect of PectaSol and Doxorubicin on viability, cell cycle arrest and apoptosis in DU-145 and LNCaP prostate cancer cell lines. *Cell Biol. Int.* **36**, 601-610 (2012).

7. Chou, M. H., Hsieh, Y. C., Huang, C. W., Chen, P. H., Chan, S. P., Tsao, Y. P. *et al.* BCAS2 Regulates Delta-Notch Signaling Activity through Delta Pre-mRNA Splicing in Drosophila Wing Development. *PLoS One* **10**, e0130706 (2015).

Fig. S1

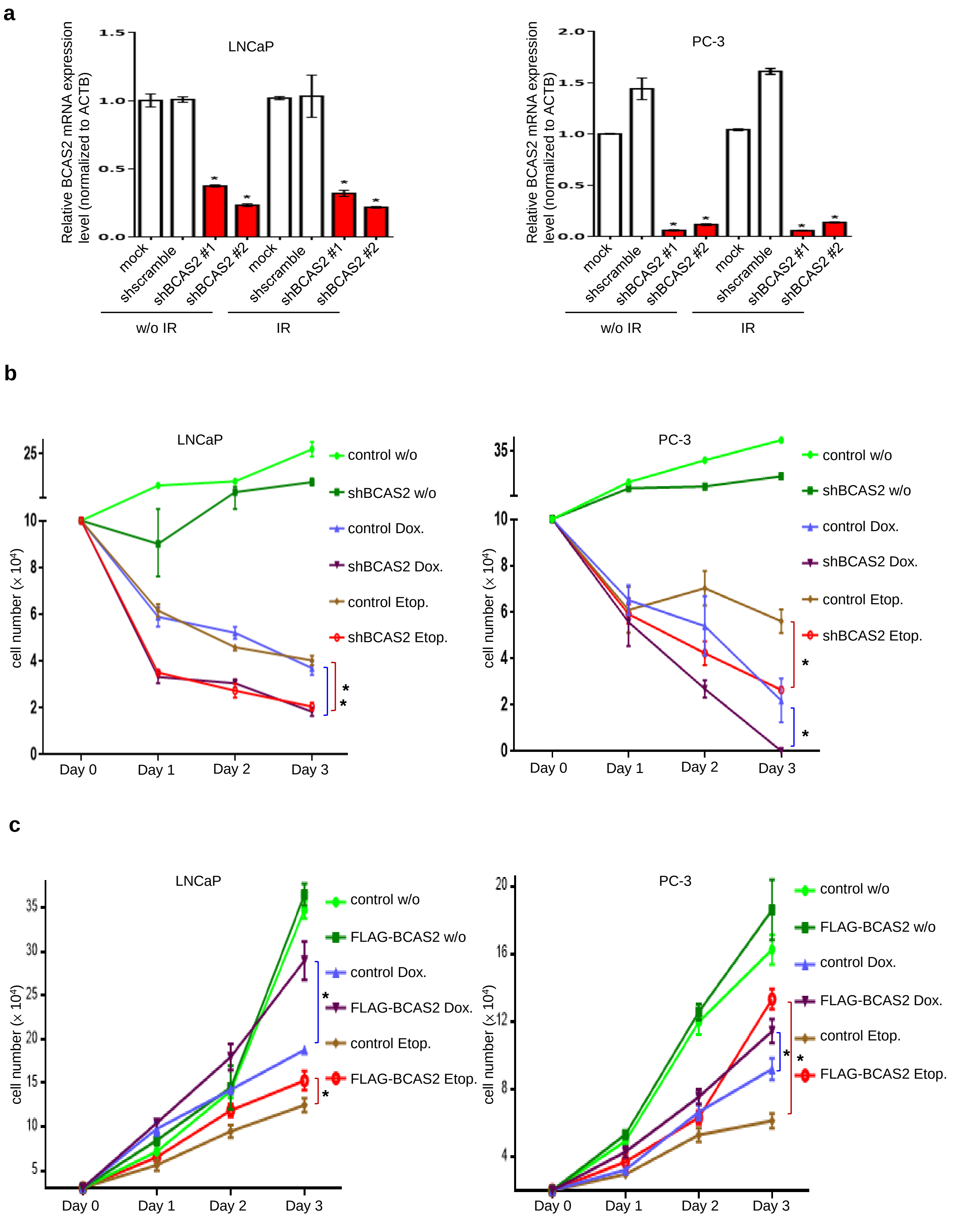
a
LNCaP
Relative BCAS2 mRNA expression level (normalized to ACTB)
1.5
1.0
0.5
0.0
mock
shscramble
shBCAS2 #1
shBCAS2 #2
mock
shscramble
shBCAS2 #1
shBCAS2 #2
w/o IR
IR
PC-3
2.0
1.5
1.0
0.5
0.0
b
LNCaP
cell number (× 10⁴)
Day 0
Day 1
Day 2
Day 3
control w/o
shBCAS2 w/o
control Dox.
shBCAS2 Dox.
control Etop.
shBCAS2 Etop.
PC-3
c
LNCaP
control w/o
FLAG-BCAS2 w/o
control Dox.
FLAG-BCAS2 Dox.
control Etop.
FLAG-BCAS2 Etop.
PC-3

Fig. S2

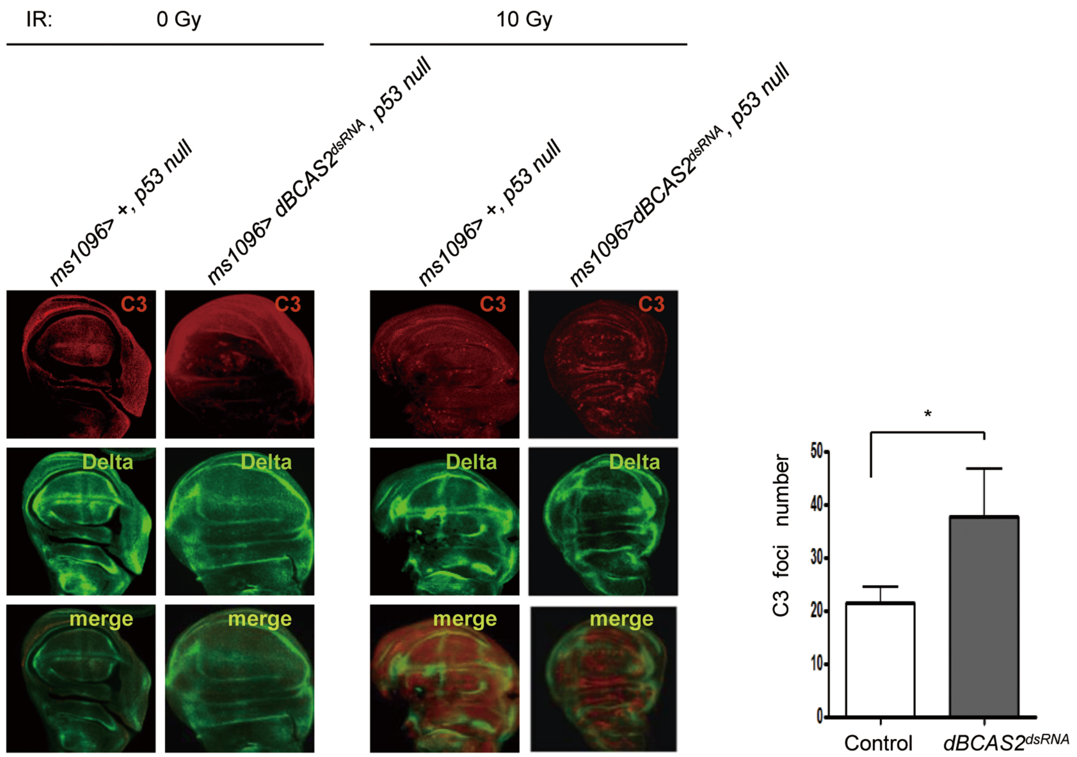
IR:
0 Gy
10 Gy
ms1096> +, p53 null
ms1096> dBCAS2dsRNA, p53 null
ms1096> +, p53 null
ms1096>dBCAS2dsRNA, p53 null
C3
Delta
merge
*
C3 foci number
0
10
20
30
40
50
Control
dBCAS2dsRNA

Fig. S3

a

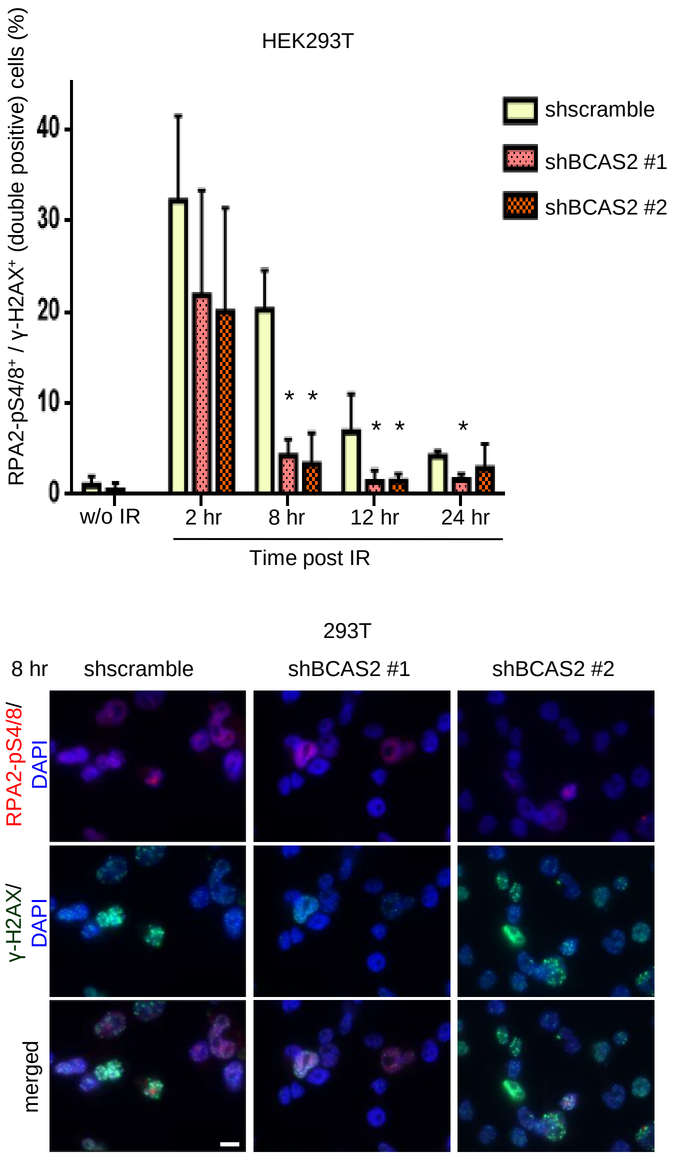
HEK293T
RPA2-pS4/8⁺ / γ-H2AX⁺ (double positive) cells (%)
0
10
20
30
40
shscramble
shBCAS2 #1
shBCAS2 #2
* *
* *
*
w/o IR
2 hr
8 hr
12 hr
24 hr
Time post IR
293T
8 hr
shscramble
shBCAS2 #1
shBCAS2 #2
RPA2-pS4/8/
DAPI
γ-H2AX/
DAPI
merged

b

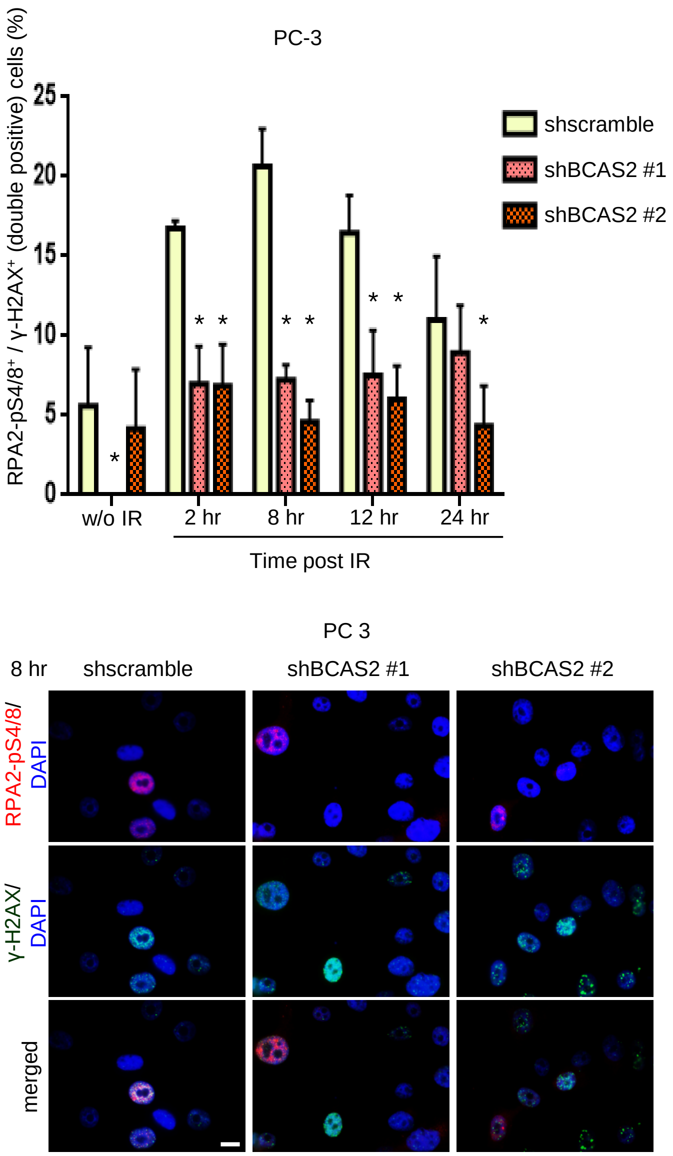
PC-3
RPA2-pS4/8⁺ / γ-H2AX⁺ (double positive) cells (%)
0
5
10
15
20
25
shscramble
shBCAS2 #1
shBCAS2 #2
*
* *
* *
* *
*
w/o IR
2 hr
8 hr
12 hr
24 hr
Time post IR
PC 3
8 hr
shscramble
shBCAS2 #1
shBCAS2 #2
RPA2-pS4/8/
DAPI
γ-H2AX/
DAPI
merged

Fig. S4

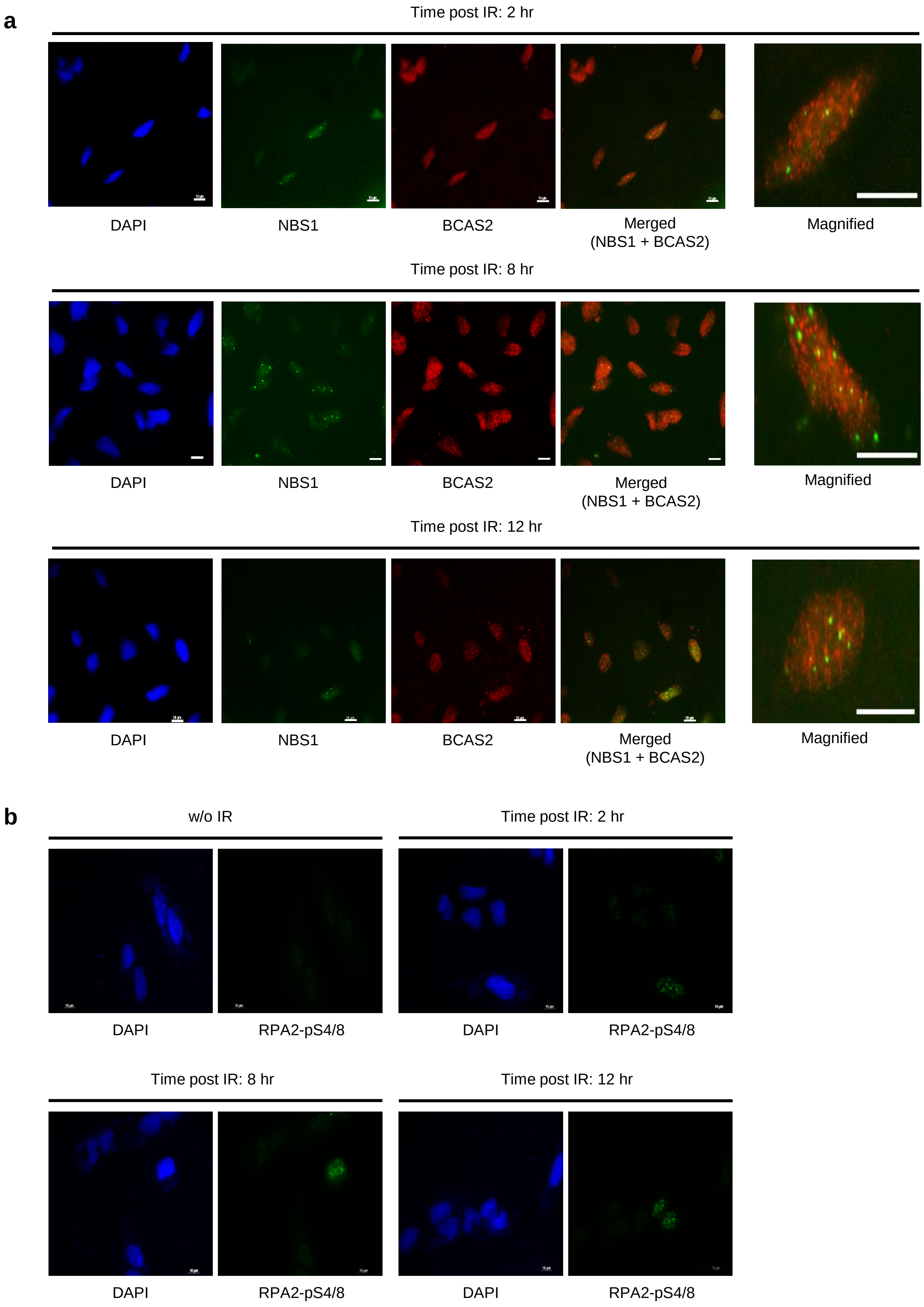
a
Time post IR: 2 hr
DAPI
NBS1
BCAS2
Merged
(NBS1 + BCAS2)
Magnified
Time post IR: 8 hr
DAPI
NBS1
BCAS2
Merged
(NBS1 + BCAS2)
Magnified
Time post IR: 12 hr
DAPI
NBS1
BCAS2
Merged
(NBS1 + BCAS2)
Magnified
b
w/o IR
DAPI
RPA2-pS4/8
Time post IR: 2 hr
DAPI
RPA2-pS4/8
Time post IR: 8 hr
DAPI
RPA2-pS4/8
Time post IR: 12 hr
DAPI
RPA2-pS4/8

Fig. S5

a

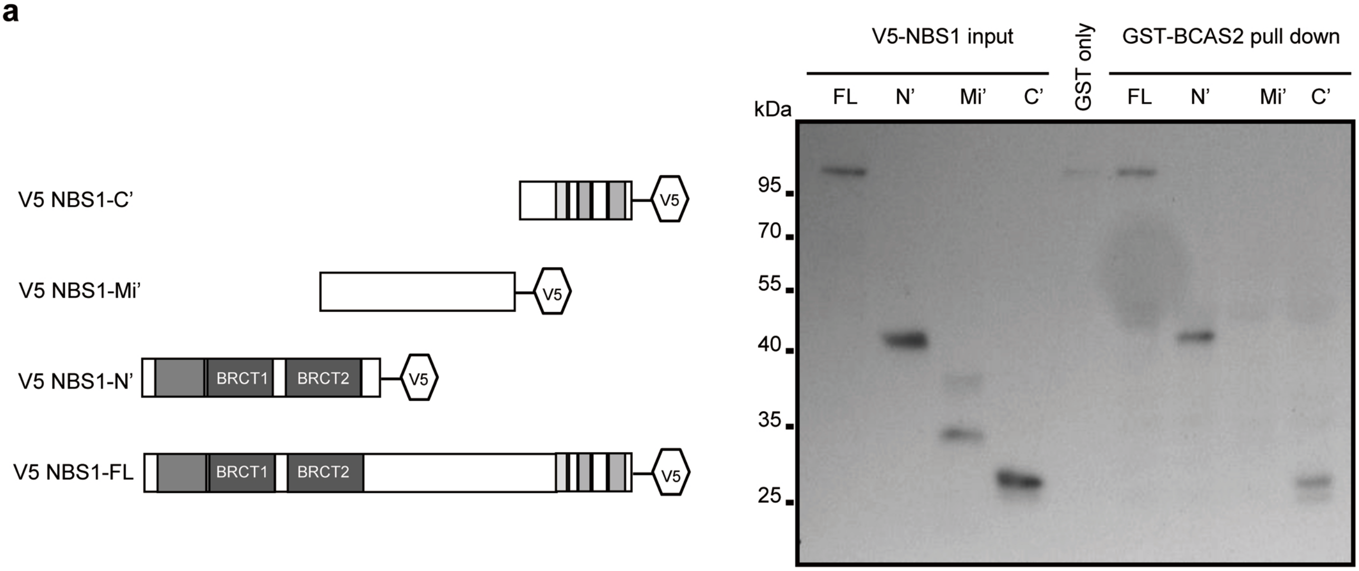
V5-NBS1 input
GST only
GST-BCAS2 pull down
kDa
FL
N'
Mi'
C'
FL
N'
Mi'
C'
95
70
55
40
35
25
V5 NBS1-C'
V5 NBS1-Mi'
V5 NBS1-N'
V5 NBS1-FL
BRCT1
BRCT2
V5

b

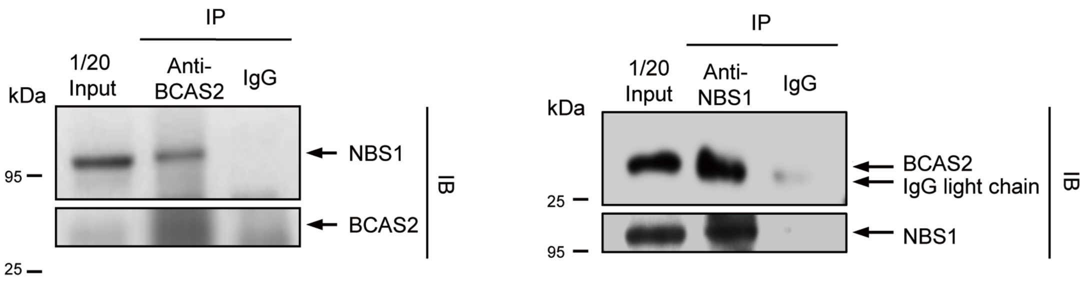
IP
1/20 Input
Anti-BCAS2
IgG
kDa
95
25
NBS1
BCAS2
IB
IP
1/20 Input
Anti-NBS1
IgG
kDa
25
95
BCAS2
IgG light chain
NBS1
IB

Fig. S6

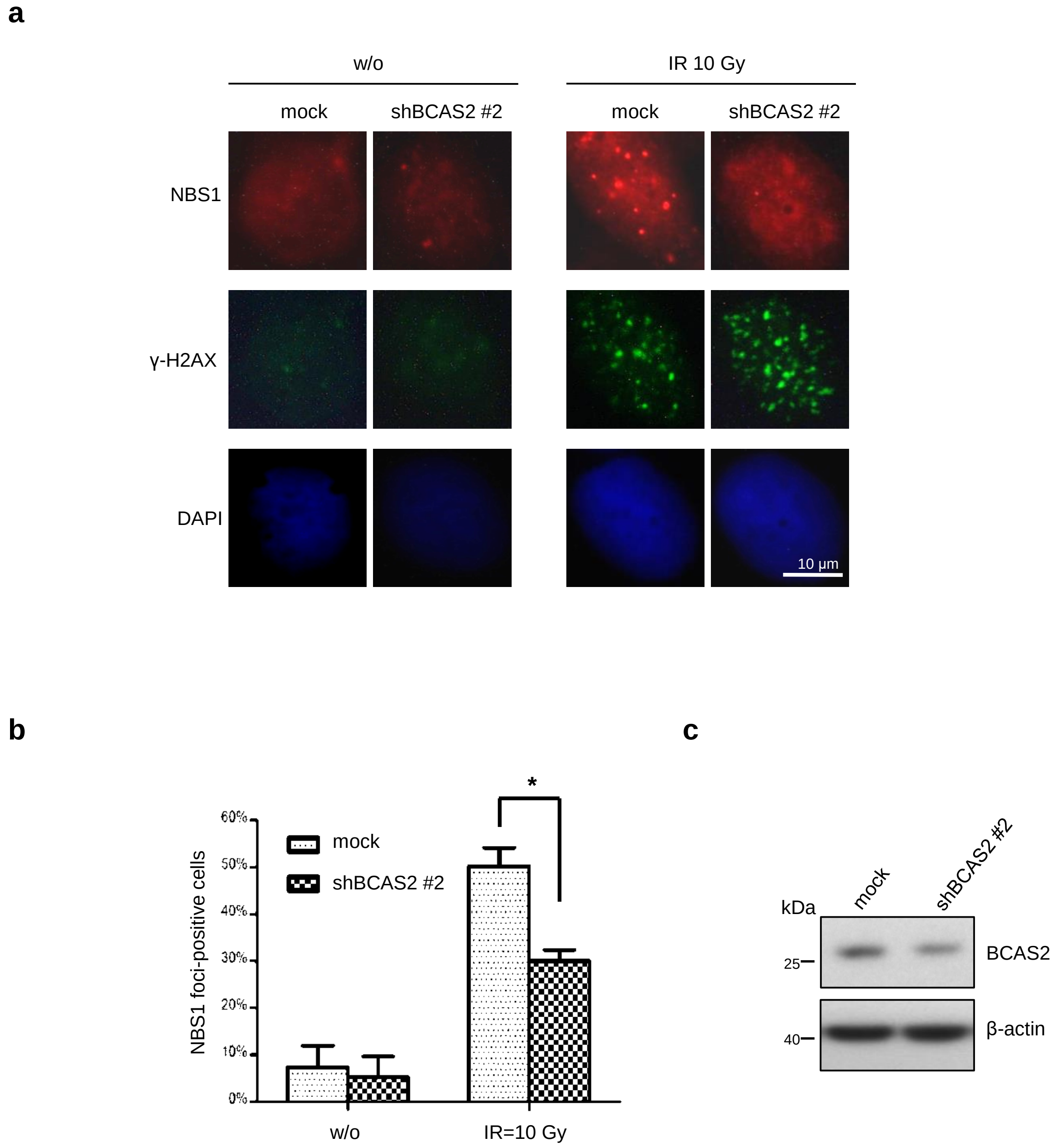
a
w/o
IR 10 Gy
mock
shBCAS2 #2
mock
shBCAS2 #2
NBS1
γ-H2AX
DAPI
10 μm
b
*
60%
50%
40%
30%
20%
10%
0%
NBS1 foci-positive cells
mock
shBCAS2 #2
w/o
IR=10 Gy
c
kDa
mock
shBCAS2 #2
25
40
BCAS2
β-actin

Fig. S7

a

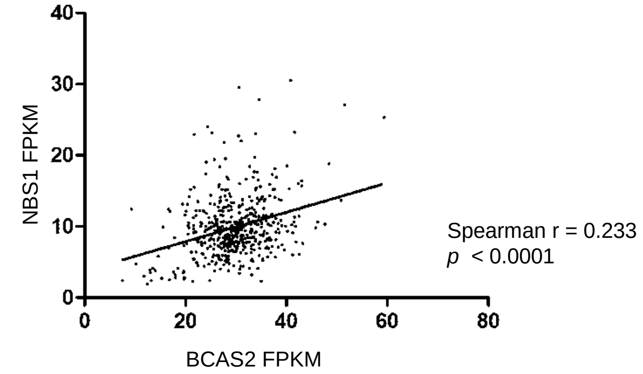

NBS1 FPKM
40
30
20
10
0
0
20
40
60
80
BCAS2 FPKM
Spearman r = 0.233
p < 0.0001

b

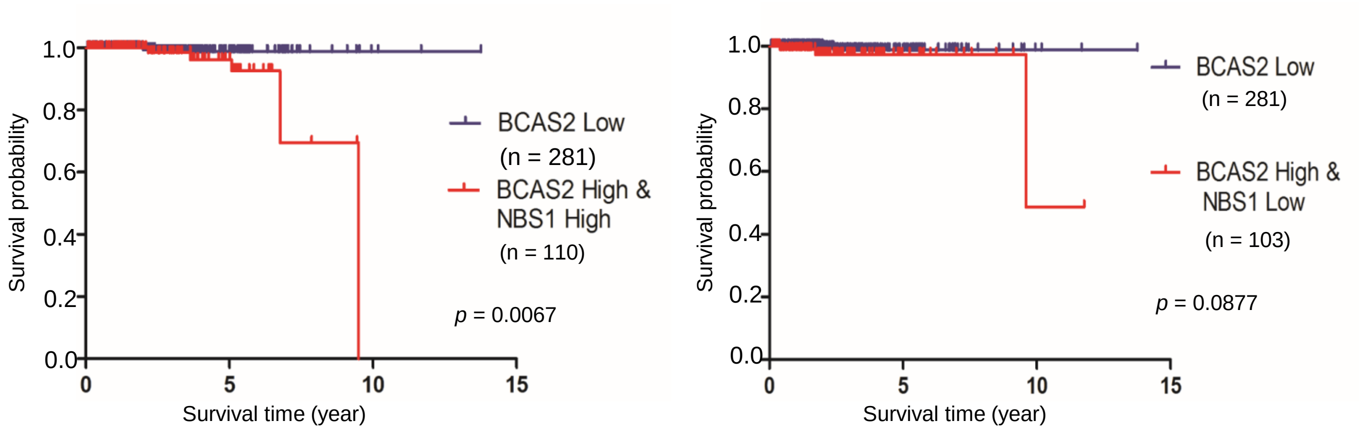

Survival probability
1.0
0.8
0.6
0.4
0.2
0.0
0
5
10
15
Survival time (year)
BCAS2 Low
(n = 281)
BCAS2 High &
NBS1 High
(n = 110)
p = 0.0067
Survival probability
1.0
0.8
0.6
0.4
0.2
0.0
0
5
10
15
Survival time (year)
BCAS2 Low
(n = 281)
BCAS2 High &
NBS1 Low
(n = 103)
p = 0.0877
